# Saturation mutagenesis of α-synuclein reveals monomer fold that modulates aggregation

**DOI:** 10.1126/sciadv.adh3457

**Published:** 2023-10-27

**Authors:** Julita Chlebowicz, William Russ, Dailu Chen, Anthony Vega, Steven Vernino, Charles L. White, Josep Rizo, Lukasz A. Joachimiak, Marc I. Diamond

**Affiliations:** ^1^ Center for Alzheimer's and Neurodegenerative Diseases, University of Texas Southwestern Medical Center, Dallas, TX, USA.; ^2^ Evozyne Inc., Chicago, IL, USA.; ^3^ Lyda Hill Department of Bioinformatics, University of Texas Southwestern Medical Center, Dallas, TX, USA.; ^4^ Department of Neurology, University of Texas Southwestern Medical Center, Dallas, TX, USA.; ^5^ Peter O'Donnell Jr. Brain Institute, University of Texas Southwestern Medical Center, Dallas, TX, USA.; ^6^ Department of Pathology, University of Texas Southwestern Medical Center, Dallas, TX, USA.; ^7^ Department of Biophysics, University of Texas Southwestern Medical Center, Dallas, TX, USA.; ^8^ Department of Biochemistry, University of Texas Southwestern Medical Center, Dallas, TX, USA.; ^9^ Department of Pharmacology, University of Texas Southwestern Medical Center, Dallas, TX, USA.

## Abstract

α-Synuclein (aSyn) aggregation underlies neurodegenerative synucleinopathies. aSyn seeds are proposed to replicate and propagate neuronal pathology like prions. Seeding of aSyn can be recapitulated in cellular systems of aSyn aggregation; however, the mechanism of aSyn seeding and its regulation are not well understood. We developed an mEos-based aSyn seeding assay and performed saturation mutagenesis to identify with single-residue resolution positive and negative regulators of aSyn aggregation. We not only found the core regions that govern aSyn aggregation but also identified mutants outside of the core that enhance aggregation. We identified local structure within the N terminus of aSyn that hinders the fibrillization propensity of its aggregation-prone core. Based on the screen, we designed a minimal aSyn fragment that shows a ~4-fold enhancement in seeding activity and enabled discrimination of synucleinopathies. Our study expands the basic knowledge of aSyn aggregation and advances the design of cellular systems of aSyn aggregation to diagnose synucleinopathies based on protein conformation.

## Teaser

Mutagenesis of α-synuclein coupled with crosslinking and NMR provides insights into how local structure modulates aggregation.

## INTRODUCTION

α-Synuclein (aSyn) is a 14-kDa protein involved in synaptic vesicle processing (*1*, *2*) and soluble N-ethylmaleimide–sensitive factor attachment protein receptor (SNARE) complex assembly (*3*). It can refold to form toxic amyloid assemblies that underlie neurodegenerative synucleinopathies, including Parkinson disease (PD), limbic and diffuse Lewy body disease (LBD) (*4*), and multiple system atrophy (MSA) (*5*). In these disorders, pathology progresses along defined neural networks, and experimental evidence suggests that aSyn propagates its conformation in a prion-like manner (*6*–*8*) in which ordered aggregates escape one cell or group of cells (*9*), gain entry to connected cells, and serve as templates for their own replication. In simple cell models, exogenous assemblies will trigger the intracellular amyloid formation of aSyn protein fused to fluorescent protein tags (*10*, *11*). Such “biosensor” cells have proved useful to detect and quantify pathology from biological samples (*8*, *12*) but are not as sensitive as similar systems to detect tau seeds (*13*). Several mutations in aSyn cause dominantly inherited synucleinopathy (*14*–*21*). However, the residues or domains of aSyn that mediate its aggregation have not been systematically studied. The region composed of amino acids 61 to 95 co-aggregates with plaques of amyloid-β (*22*, *23*), but the precise roles of subdomains, and the N and C termini of the protein, are unknown.

To address these questions, we performed a saturation mutagenesis screen in a human embryonic kidney (HEK) 293T aSyn biosensor reporter cell line and characterized mutants that increased or decreased seeding propensity. We followed up these experiments with truncation analyses, cross-linking with mass spectrometry (MS), and nuclear magnetic resonance (NMR) spectroscopy. These experiments provided insight into how local structure controls aSyn propensity for aggregation. We propose a model in which the N terminus of aSyn modulates the fibrillization propensity of its aggregation-prone core.

## RESULTS

### Saturation mutagenesis screen reveals aSyn domains that drive fibril formation

**Fig. 1.**
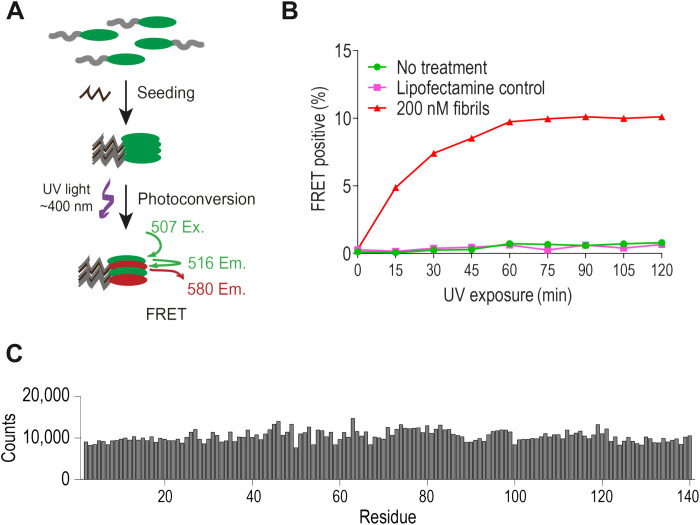
Creation of aSyn-mEos FRET biosensor cells and library sequencing. (**A**) Green fluorescent mEos proteins are partially photoconverted to red with UV light. This forms a green-red FRET pair upon aggregation induced by recombinant fibrils. Ex., excitation; Em., emission. (**B**) Detection of aggregates in aSyn(A53T)-mEos biosensor cells seeded with 200 nM recombinant aSyn(WT) fibrils or vehicle (Lipofectamine 2000) after increasing exposure to UV light. (**C**) Counts for each mutated aSyn residue obtained from deep sequencing of the input saturation library.

We sought to create a saturation screen that would rely on intracellular expression of one mutant aSyn per cell. Thus, we developed an aSyn(A53T)-mEos stable cell line similar to that which we have previously reported (*12*). Treatment with ultraviolet (UV) light converts mEos emission from green to red, enabling red-green fluorescence resonance energy transfer (FRET) when both forms of the protein are in close proximity (Fig. 1A) (*24*, *25*). We optimized the UV conversion protocol for a maximal FRET signal, which reached 10% in cells treated with 200 nM wild-type (WT) aSyn fibrils (Fig. 1B).

**Fig. 2.**
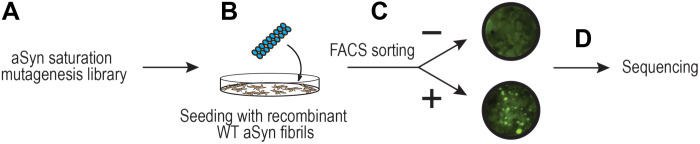
Saturation mutagenesis screen workflow. (**A**) A saturation mutagenesis library of 2780 single-point aSyn mutants was expressed in HEK293T cells. (**B**) Recombinant aSyn fibrils were transfected into cells to induce aggregation (seeding). (**C**) FRET-FACS was used to separate seeded and unseeded cells. (**D**) DNA from each pool was extracted and sequenced.

We used sequential degenerate codon primers to construct a saturation library of single-point mutants of aSyn at every position. The pooled library encoded 2780 aSyn variants C-terminally fused to the mEos tag (aSyn-mEos). Deep sequencing of the input library revealed consistent distribution of mutations across the length of the gene and the absence of multiple mutations within the same construct (Fig. 1C). We then transduced HEK293T cells with the library at a multiplicity of infection of <0.3 to reduce multiple integration events in a cell (Fig. 2A). Cells were then transfected with sonicated recombinant aSyn(WT) fibrils (200 nM) using Lipofectamine 2000 to induce seeding of the intracellular aSyn (Fig. 2B and fig. S1, E and F). After harvesting and fixation, we photoconverted approximately 80% of cells for both green and red mEos using UV illumination for 1 hour. We then used FRET-based fluorescence-activated cell sorting (FACS) to sort the cells into two pools: (i) FRET-positive (aggregate-containing) and (ii) FRET-negative (no aggregates) (Fig. 2C). We extracted DNA from each population, followed by deep sequencing to detect residues that modulated aSyn aggregation (Fig. 2D).

**Fig. 3.**
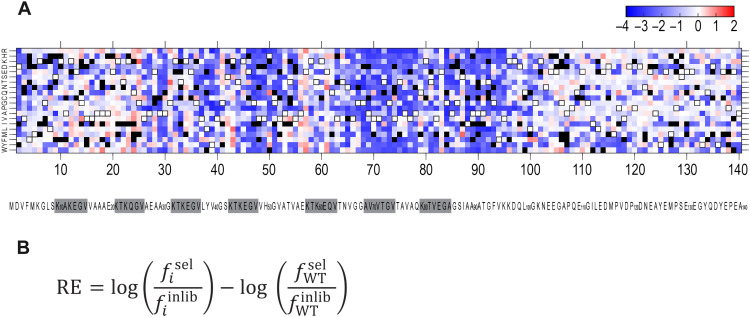
Saturation mutagenesis screen of aSyn. (**A**) RE of each point aSyn mutant in the FRET-positive population of cells treated with 200 nM aSyn(WT) fibrils. The horizontal axis shows aSyn residues 2 to 140; the left vertical axis indicates missense amino acid substitution in the following order: R, H, K, D, E, S, T, N, Q, C, G, P, A, V, I, L, M, F, Y, and W. The scale bar indicates the log_10_ enrichment. White pixels with a black frame represent WT residues. Positive RE (red color scale) represents mutants overrepresented versus WT, presumably with higher aggregation propensity. Negative RE (blue color scale) represents mutants underrepresented versus WT, presumably with lower aggregation propensity. White pixels without a frame represent mutants similar to WT. Black pixels represent mutants not detected sufficiently for an accurate count and thus excluded from further analysis. The WT sequence of aSyn is included below the matrix. Imperfect repeats are highlighted in gray. (**B**) Formula used to calculate relative enrichment. *f_i_*^sel^ = frequency of a mutant *i* in the selected population; *f_i_*^inlib^ = frequency of a mutant *i* in the input library; *f*_WT_^sel^ = frequency of the WT protein sequence (reference sequence) in the selected population; *f*_WT_^inlib^ = frequency of the WT protein sequence (reference sequence) in the input library.

To identify which mutants decreased or increased aSyn seeding propensity, we calculated the relative enrichment (RE) for each variant within sorted populations (Fig. 3, A and B). This revealed regions sensitive to virtually any mutation (Fig. 3A). We also observed a consistent inhibitory effect of a proline substitution at most positions along the sequence. We rediscovered some but not all disease-linked residues in the screen [such as inhibitory G51D (*26*) and A53E (*27*) with RE −2.64 and RE −0.22, or aggregation-prone H50Q (*28*), A53V (*29*), and E83Q (*30*) with RE 1.00, RE 0.34, and RE 0.17, respectively]. On the basis of stop codon frequencies, we calculated a 3.6% false discovery rate (FDR), most likely associated with the technical limitations of FACS. We detected no de-enrichment from any mutant under basal conditions to indicate toxicity (fig. S1, A and B).

**Fig. 4.**
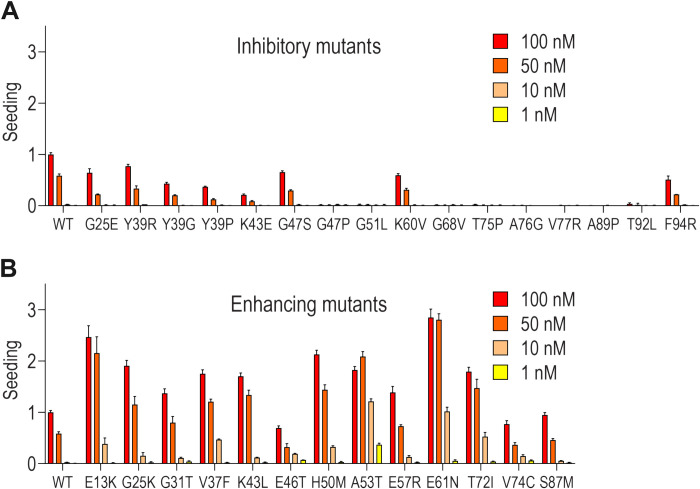
Retesting mutants that modulate aSyn aggregation. aSyn variants identified as inhibitory (**A**) or aggregation prone (**B**) were picked and cloned individually as aSyn-CFP/YFP polyclonal biosensors for comparison to aSyn(WT). Cells without treatment or treated with vehicle (Lipofectamine 2000) did not contain aggregates (fig. S3A). Analysis was performed after 48-hour incubation. Seeding efficiency was normalized to the aSyn(WT) treated with 100 nM recombinant aSyn(WT) fibrils. Error bars = SD (*n* = 3 technical replicates).

To test these mutations for seeding potential, we picked 16 de-enriched and 13 enriched mutants, cloned them upstream of cyan or yellow fluorescent protein (aSyn-CFP or aSyn-YFP), and created high-expressing polyclonal biosensor lines. The literature suggests that these tags do not have any substantial effect on aSyn aggregation (*31*, *32*), especially when placed on the C terminus, which remains flexible and disordered in the case of aSyn. We then compared seeding to aSyn(WT) or aSyn(A53T) treated with a range of aSyn fibril concentrations (Fig. 4, A and B). Each mutant had a different dose-response curve to recombinant fibrils. Inhibitory mutants localized at residues 65 to 78 were the strongest across all seed concentrations. We observed increased seeding for aSyn(E13K) and aSyn(E61N), equal to or higher than aSyn(A53T). Overall, secondary testing confirmed the results of the primary screen and indicated that the sorting predicted mutations with differential effects on aggregation.

### Saturation mutagenesis identifies inhibitory and aggregation-prone hotspots

**Fig. 5.**
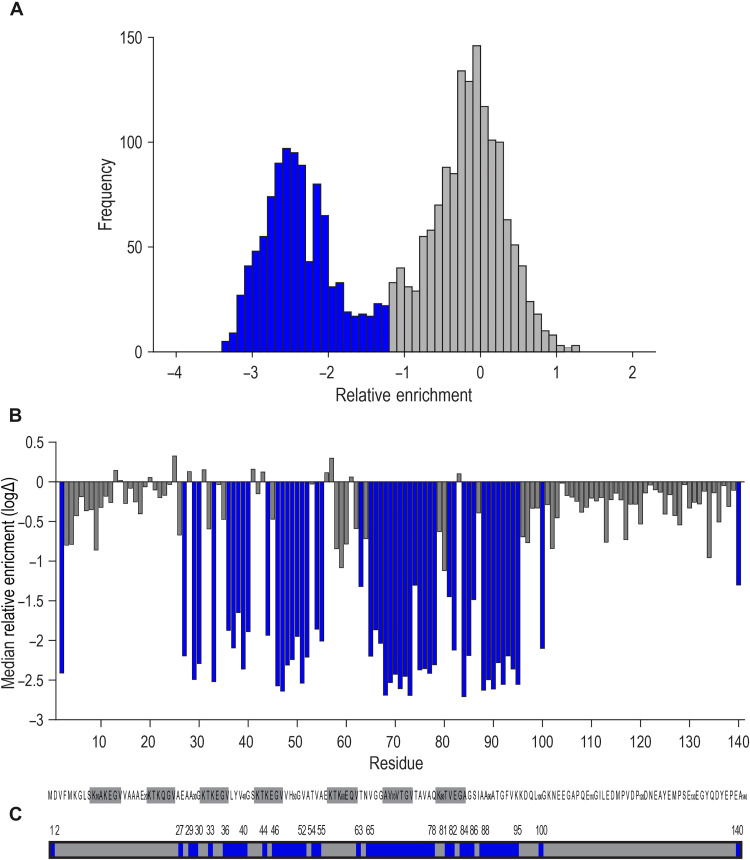
aSyn domains that drive seeding. (**A**) Histogram representing the frequency distribution of RE within the mutagenesis data (data for stop codons and missing data excluded). Residues with RE equal or lower than calculated Otsu threshold (−1.2152) were marked as blue bars and identified as aSyn domains promoting seeded aggregation. (**B**) Median RE (mRE) for each mutated aSyn residue (data for stop codons and missing data excluded). Blue bars indicate residues with mRE equal to or lower than −1.2152; gray bars indicate residues with mRE higher than the threshold. (**C**) Residues important for aSyn seeding are marked in blue: 2, 27, 29 to 30, 33, 36 to 40, 44, 46 to 52, 54 to 55, 63, 65 to 78, 81 to 82, 84 to 86, 88 to 95, 100, and 140.

**Fig. 6.**
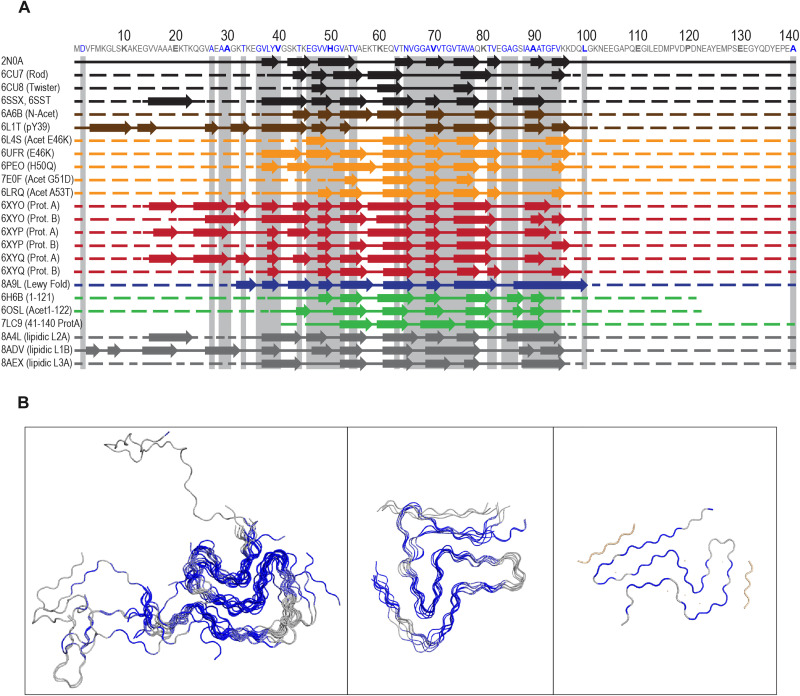
Localization of aSyn domains across distinct structures. (**A**) Comparison of secondary structures from multiple available ssNMR and cryo-EM datasets (full comparison in fig. S2D). Arrows indicate localization of β sheets, a continuous line indicates regions with a core structure, and a dashed line indicates aSyn “fuzzy coat.” Black: Structures of recombinant aSyn(WT) fibrils; brown: structures of recombinant aSyn(WT) fibrils containing posttranslational modifications; yellow: structures of recombinant aSyn containing disease-linked mutations; red: aSyn structures derived from MSA brain; dark blue: aSyn structure derived from LBD or PD brain; green: truncated recombinant aSyn fibrils (*44*–*46*); gray: recombinant aSyn fibrils induced by lipids. Protein Data Bank (PDB) ID for each structure is indicated on the left. Gray horizontal stripes indicate localization of aSyn residues driving its aggregation. β sheets were assessed using a dss command in PyMOL. (**B**) Alignment of monomers derived from available aSyn fibril structures shows three distinct common fibril folds (*34*–*42*, *44*, *45*) [the third one is PDB ID 8A9L (*43*)] and in blue localization of aSyn residues important for its aggregation.

To systematically assess aSyn domains most sensitive to any substitution, we calculated the median RE across all mutations for each residue (missing data and data for stop codons were excluded) and applied a variance-based Otsu thresholding method (*33*) to split data into two classes. This technique seeks to maximize the intervariance while minimizing the intravariance in a bimodal population. We classified as strong inhibitors all mutations with a median value equal to or less than the threshold (−1.2152) (Fig. 5, A and B). This revealed aSyn domains important for aggregation that included the following residues: 2, 27, 29 to 30, 33, 36 to 40, 44, 46 to 52, 54 to 55, 63, 65 to 78, 81 to 82, 84 to 86, 88 to 95, 100, and 140 (Fig. 5, B and C). To place these domains within the context of prior studies of aSyn, we mapped them into available solid-state NMR (ssNMR) or cryogenic electron microscopy (cryo-EM) structures (Fig. 6A and fig. S2D). Some mutational hotspots overlapped with β-strand regions. We next aligned monomer conformations (i.e., single filament layer) from available aSyn fibril structures (*34*–*45*), which revealed three main aSyn fibril folds. The core amyloidogenic regions identified in our mutagenesis screen form interactions within each fold using different geometries (Fig. 6B). These data were consistent with amyloid motifs that stabilize aSyn monomer folds as critical for amyloid formation.

**Fig. 7.**
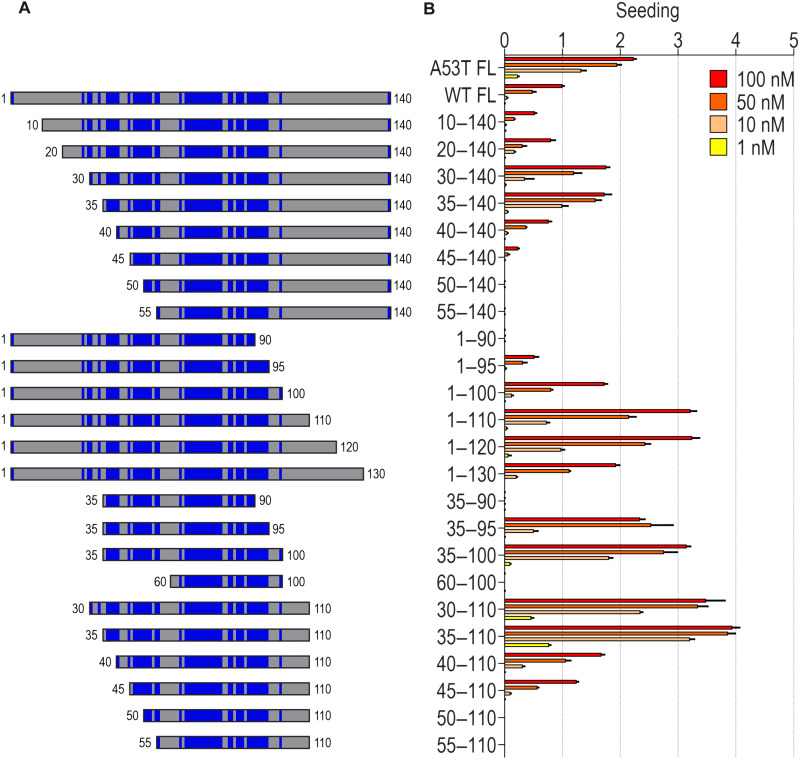
Required domains and inhibitory elements for aSyn aggregation. (**A**) aSyn truncation constructs. Domains/residues driving aSyn aggregation identified by mutagenesis are marked in blue: 2, 27, 29 to 30, 33, 36 to 40, 44, 46 to 52, 54 to 55, 63, 65 to 78, 81 to 82, 84 to 86, 88 to 95, 100, and 140. (**B**) aSyn truncations fused to CFP/YFP were overexpressed in HEK293T cells, treated with increasing amounts of recombinant aSyn(WT) fibrils, and analyzed after 48 hours. Cells without treatment or treated with vehicle (Lipofectamine 2000) did not contain aggregates (fig. S3A). Seeding efficiency was normalized to aSyn(WT) treated with 100 nM recombinant aSyn(WT) fibrils. Error bars = SD (*n* = 3 technical replicates).

The mutagenesis predicted an important role for the aSyn core in seeded aggregation. Thus, we tested the influence of N- and C-terminal sequences on aggregation by truncating aSyn-CFP/YFP fusions and stably overexpressing them in HEK293T cells (Fig. 7, A and B). Deletion of amino acids 1 to 19 decreased aSyn aggregation in comparison with the full length, but removal of amino acids 1 to 29 and amino acids 1 to 34 increased aggregation. Removal of amino acids 1 to 54 blocked aggregation entirely. We observed similar effects with C-terminal truncations, consistent with observations by others (*46*–*48*). Removal of amino acids 101 to 140 increased aggregation in comparison with the full length. Deletion of amino acids 91 to 140 blocked aSyn aggregation. We observed a maximal effect with both N- and C-terminal truncations, leaving amino acids 35 to 110 (Fig. 7, A and B). Addition of the disease-linked mutations (E46K, H50Q, and A53T) slightly increased seeding capacity of the truncated sequence (fig. S3A).

### Mutations create differential responses to MSA, PD, and LBD

**Fig. 8.**
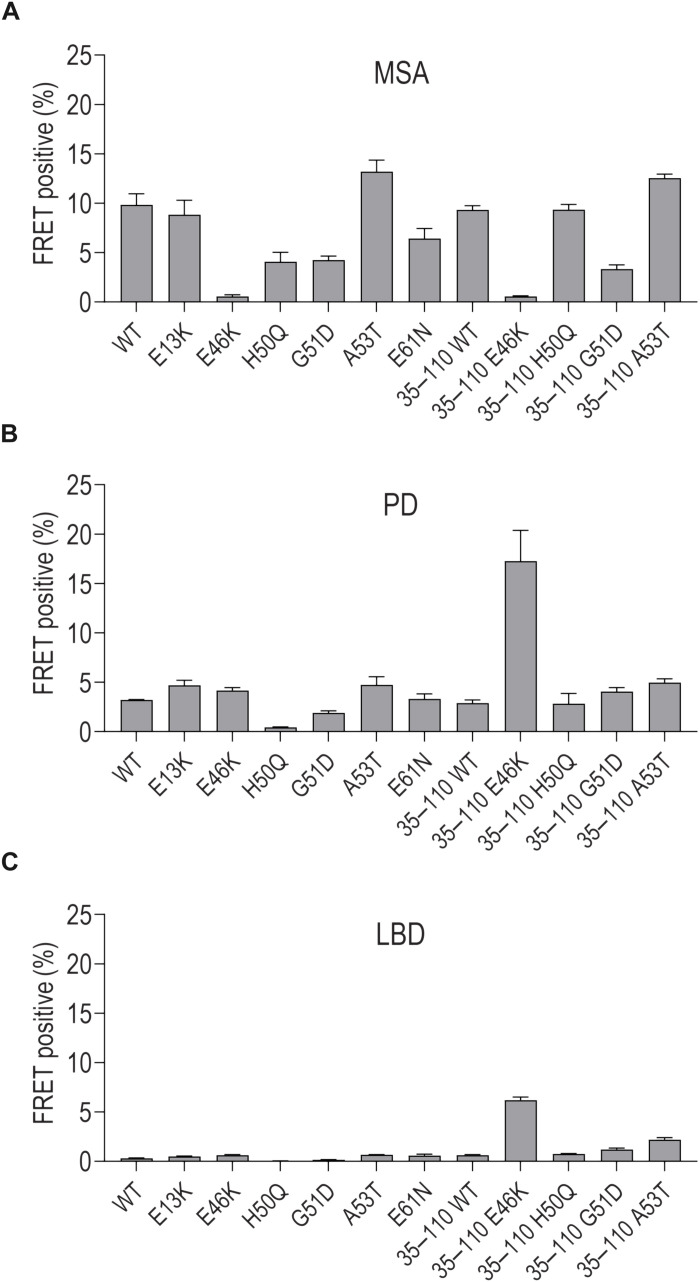
A biosensor expressing truncated E46K aSyn identifies LBD fibrils with high sensitivity. Seeding of 1 μg of aSyn fibrils extracted from MSA (**A**), 5 μg of aSyn fibrils extracted from PD (**B**), and 15 μg of aSyn fibrils extracted from LBD (**C**) brain tissue on a panel of monoclonal HEK293T biosensors (full dataset shown in fig. S3B). Cells without treatment or treated with a vehicle (Lipofectamine 2000) (fig. S3A) or negative control fibrils (100 nM tau, Aβ40, and Aβ42 or 5 μg of sarkosyl-insoluble material from a control healthy brain) did not contain aggregates. Analysis was performed after 72-hour incubation. Error bars = SD (*n* = 3 technical replicates).

The prion hypothesis predicts differential seeding efficiencies of strains based on the amino acid composition of the monomer. To test this idea for aSyn, we purified sarkosyl-insoluble fibrils from MSA, PD, and LBD brain tissue and transfected a panel of various biosensors (Fig. 8, A to C). MSA fibrils efficiently seeded all except for the E46K mutant, as has been previously reported (*8*). By contrast, PD and LBD fibrils efficiently seeded this mutant, especially the truncated version (amino acids 35 to 110). Two truncated biosensors discriminated LBD and MSA fibrils: E46K and A53T. As previously reported (*8*, *12*, *49*), we observed higher seeding capacity with homogenates from MSA versus LBD and PD. Moreover, transmission electron microscopy (TEM) of the sarkosyl-insoluble material and a Western blot (fig. S3, C to E) showed higher number of fibrils in MSA and PD and the lowest amount in the LBD material. The amount of fibrils correlated with the seeding level.

### Local structure of the N terminus suppresses aggregation

**Fig. 9.**
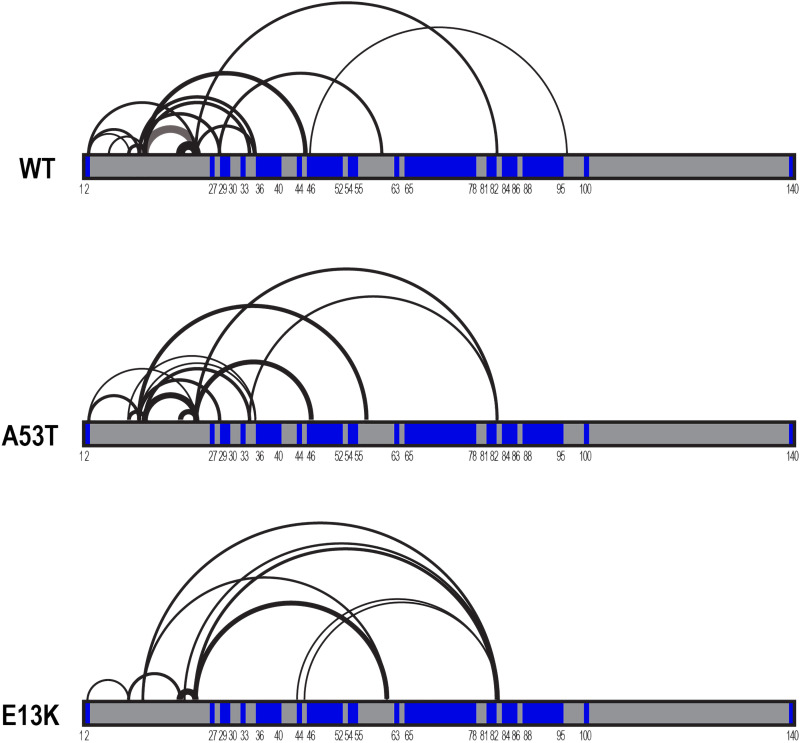
Cross-link maps of WT, A53T, and E13K aSyn. Cross-linking data for aSyn(WT), aSyn(A53T), and aSyn(E13K), performed with DMTMM in 20 mM Hepes (pH 8.0) and 100 mM NaCl. Gray bars represent aSyn monomer with identified domains driving its aggregation (blue). Loops indicate cross-linked residues, and the line thickness represents mean frequency in the dataset. All indicated cross-links were present in five of five technical replicates.

Deletion analysis suggested that the N terminus of aSyn might modulate aggregation. Cross-linking with MS (XL-MS) has proven useful not only to model protein assemblies but also to infer changes in conformations of intrinsically disordered proteins such as tau (*50*). We first used DMTMM [4-(4,6-dimethoxy-1,3,5-triazin-2-yl)-4-methyl-morpholinium chloride] as a cross-linker, which covalently cross-links lysines to acidic residues (glutamic or aspartic acids), reporting on complementary charge interactions (*51*), to understand differences between aSyn(WT) and aSyn(A53T) monomer. After cross-linking, aSyn monomer was excised from an SDS–polyacrylamide gel electrophoresis gel (fig. S4A) and trypsinized, and five sample replicates were analyzed by MS. The data were processed using the xQuest pipeline to identify high-confidence cross-links within aSyn monomers, considering only cross-links that were observed across all five replicates. It was not possible to evaluate the C terminus after amino acid 102, because trypsin digestion created a large negatively charged peptide (amino acids 103 to 140), which was undetectable in liquid chromatography–MS/MS (LC-MS/MS). We identified 31.6 and 25.8 cross-links (mean across replicates) within aSyn(WT) and aSyn(A53T), respectively (Fig. 9 and fig. S4, B to D). XL-MS indicated that aSyn(WT) contained 24.6 cross-links within the N-terminal 35 amino acids (Fig. 9 and fig. S4, B to D). We also detected long-range interactions: a single contact between amino acid 46 within the protein core and amino acid 97 in the C terminus, and additional contacts between the N terminus and the protein core (13/45, 23/83, and 28/60) (Fig. 9 and fig. S4, B to D). In the aSyn(A53T) samples, we detected fewer cross-links within the N terminus, potentially indicating relative destabilization of the local N-terminal interactions (Fig. 9 and fig. S4, B to D). aSyn(A53T) also lacked the contact between amino acid 46 within the protein core and amino acid 97 in the C terminus, consistent with greater exposure of the hydrophobic core sequences amino acids 36 to 95.

E13 was one of the most highly cross-linked residues, and aSyn(E13K) aggregated more efficiently (Fig. 4). We hypothesized that this could derive from disruption of electrostatic interactions of E13 with a cluster of lysines in the N terminus. XL-MS of the aSyn(E13K) monomer revealed almost complete loss of N-terminal interactions (6.6 cross-links), despite efficient cross-linking to K13, suggesting perturbation of local structure (Fig. 9 and fig. S4, B to D). aSyn(E13K) also lacked cross-links between amino acid 46 and amino acid 97, similar to aSyn(A53T). By contrast, another interactions appeared between the N terminus and the aSyn core.

### NMR reveals local structure at the N terminus of aSyn

**Fig. 10.**
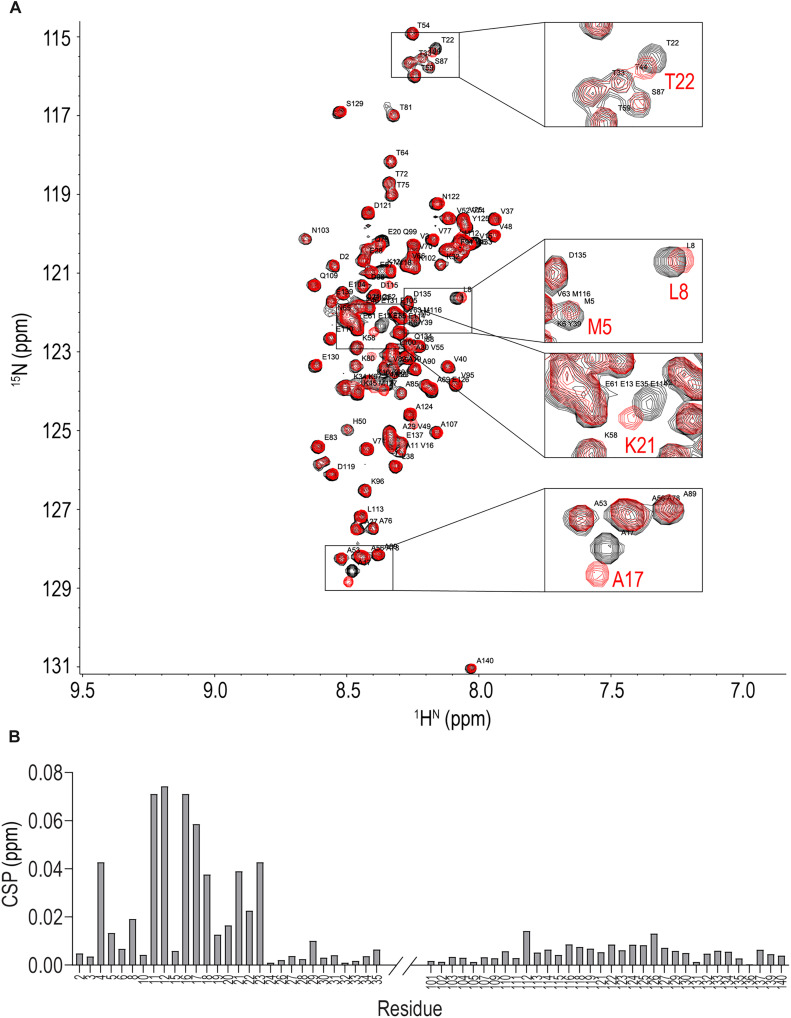
^1^H-^15^N HSQC spectral changes indicate N-terminal local structure perturbed by E13K. (**A**) ^1^H-^15^N HSQC spectra of aSyn(WT) (black) and aSyn(E13K) (red) recorded at 15°C and 30 μM concentration in 20 mM Hepes (pH 7.4) and 100 mM NaCl. Full spectra shown in fig. S5A. (**B**) CSPs caused by the E13K mutation in the ^1^H-^15^N HSQC spectra shown in (A) (formula used for calculation is described in Materials and Methods). Shifts for residues with lower assignment confidence were excluded from the graph and calculations. Average CSP = 0.00997 ppm. Full data are shown in fig. S5B.

Models based on XL-MS predicted that aSyn(E13K) has a different conformation within the N terminus. To test that hypothesis, we analyzed ^15^N-labeled recombinant aSyn(WT) and aSyn(E13K) by NMR spectroscopy. We collected heteronuclear single quantum coherence (HSQC) of aSyn(WT) and aSyn(E13K) (Fig. 10A) and used the previously described chemical shift assignments of the WT protein (*52*) to interpret the spectral changes caused by the E13K mutation. Spectra were sufficiently well dispersed to allow unambiguous assignment of 123 cross-peaks to specific residues among 135 (excluding 5 prolines) present in aSyn at pH 7.4 and 120 cross-peaks at pH 6.0 (Fig. 10, A and B, and fig. S5, A to D and F to I). We observed substantial chemical shift perturbations (CSPs) for amino acids 4 to 23 in aSyn(E13K) compared to aSyn(WT) (Fig. 10B and fig. S5, A to D and F to I). Together with the deletion analysis and XL-MS studies, these data indicated that the E13K substitution perturbs local structure, likely inactivating the inhibitory function of the N terminus.

## DISCUSSION

We have used saturation mutagenesis, deletion analysis, XL-MS, and NMR spectroscopy to identify the sequence determinants of aSyn aggregation. We began by identifying aSyn residues enriched or de-enriched in biosensor cells seeded with recombinant aSyn fibrils. A selected number of mutants were retested and confirmed, validating the primary screen and allowing identification of residues important for aggregation. These mapped onto regions predicted to be important based on cryo-EM of multiple aSyn fibrils. Deletion analysis revealed an inhibitory role for both the N and C termini and allowed us to augment the sensitivity of aSyn biosensors by at least fourfold. In addition, we identified mutants that added discriminatory power to aSyn biosensors. XL-MS suggested that the N terminus has local structure, and the E13K mutation suggested that disruption of local electrostatic interactions might underlie increased seeding. We tested this idea using NMR spectroscopy. This mutation perturbed local structure in the region beyond that expected in the immediate vicinity of the mutated residue. We conclude that transient local structure in the aSyn N terminus mediates interactions that inhibit aggregation. These studies may enable understanding of cellular controls of aSyn aggregation and provide new tools for the analysis of synucleinopathies.

### Core domains involved in aggregation

Saturation mutagenesis revealed aSyn regions that facilitate aggregation. These included the following amino acids: 2, 27, 29 to 30, 33, 36 to 40, 44, 46 to 52, 54 to 55, 63, 65 to 78, 81 to 82, 84 to 86, 88 to 95, 100, and 140. Many key residues overlapped with the putative aSyn fibril core (61 to 95) (*22*, *23*). The domain sequences contain not only predominantly hydrophobic residues valines (V) and alanines (A) but also flexible glycines (G) and polar threonines (T) (28, 22, 18, and 14%, respectively) with high β sheet propensity. They are expected to be involved in the hydrophobic interactions across the fibril, crucial for maintaining the fold of the amyloid core. Regions where glycines are substituted could lose backbone flexibility causing potentially a dramatic change in the aggregation propensity. On the other hand, the intervening sequences have high content of charged lysines (K), glutamic acids (E), and polar glutamines (Q) (34.6, 19.2, and 11.5%, respectively, within the region 28 to 99). Not only they could play a role of “aggregation gatekeepers” (*53*, *54*) but their long side chains could be easily replaced by almost any amino acid without steric issues. Thus, we observed a lower effect of substitutions in these regions. Most aSyn amino acid substitutions decreased aggregation, and very few clearly enhanced it. We observed no spontaneous aggregation induced by mutants within the timeframe of our experiments (fig. S1C). The domain encompassed by amino acids 65 to 78 represented an indispensable core for seeding, as mutations localized in that region completely abolished aggregation (Fig. 4A). The amino acids 71 to 82 have been proposed previously by Giasson *et al.* (*55*) as a region essential for aSyn filament assembly. Other groups have previously attempted to characterize aSyn aggregation hotspots (*56*–*58*), mainly using in silico approaches. These produced similar but not identical findings as the cell-based investigation performed here, which exploited functional analyses of single amino acid substitutions to produce a detailed empirical “aggregation map” of aSyn. Previously, Newberry *et al.* (*59*) used saturation mutagenesis to probe aSyn determinants of its toxicity in yeast and concluded that the formation of an extended helix of monomeric aSyn was responsible. However, this work did not directly probe aSyn aggregation, as was done here.

To evaluate the genetic data more effectively, we used *t*-distributed stochastic neighbor embedding (t-SNE) (*60*, *61*), a method designed to reduce dimensionality and identify mutation covariance. We observed clustering between residues within identified regions and outer sequences (fig. S2B), confirming the importance of these domains. The residue coclustering within the domains was characterized by median RE below −1.2152. Analyzing t-SNE plots for each amino acid used for substitution (fig. S2C), we noted that among all 20 amino acids, arginine most disrupted the aggregation core domains but had no effect in the regions in between or within the termini. Proline blocked aggregation when introduced throughout the core region, including intervening sequences, but had no effect within the termini. The tryptophan substitution effect was less specific to location. These effects could be explained by the long positively charged aromatic side chain in arginine, changes in geometry of the protein backbone for proline, and a large aromatic side chain for tryptophan, which might cause energetic incompatibility with tightly packed β sheets within the amyloid core. These amino acids have been previously termed aggregation gatekeepers (*53*, *54*).

### Monomer fold stability in aggregation

Our data highlighted aSyn regions important for both fibril formation and inhibition. These formed stabilizing interactions compatible with the three classes of protofilament folds observed experimentally. It remains unknown how aSyn forms structural polymorphs. Seeding of recombinant aSyn with patient material in vitro has not recovered conformations observed in disease (*62*). This suggests that other factors such as ligands or cofactors or perhaps posttranslational modifications (PTMs) may be required to promote assembly into specific structures.

Recent data suggest that tau monomer also adopts seed-competent conformations dictated by a combination of local and global rearrangements (*50*, *63*). Deletion of the N and C termini in tau promotes fibril formation and, under specific buffer conditions, predisposes to conformations observed in disease (*64*). Similarly, in aSyn, deletion of flanking elements or mutations in the N terminus (E13K) promoted aggregation. In solution, aSyn likely adopts an ensemble of conformations that rapidly exchange between pro- and anti-aggregation states. The pro-aggregation interactions in aSyn probably involve stabilizing nonpolar contacts between regions that promote aggregation, similar to those observed in the fibrillar states that compete away protective interactions (i.e., flanking regions). Thus, to control aSyn assembly into distinct structural polymorphs, it may be possible to stabilize these nonpolar interactions within a defined monomer fold. In addition, it may be possible to engineer aSyn sequences that more stably adopt a subset of pro-aggregation states that shift the folding equilibrium toward a particular structure that mediates assembly.

### Local N-terminal structure

XL-MS identified N-terminal local structure within aSyn(WT) that may inhibit aggregation. This was destabilized in case of aSyn(A53T) and almost eliminated in aSyn(E13K). XL-MS also suggested that these variants change the exposure of the hydrophobic core, which would presumably be masked by intramolecular folding. Together, these findings indicated that regulation of aSyn aggregation may hinge on exposure of its hydrophobic core [suggested also by others (*65*–*67*)] based in part on N-terminal local structure that masks it. Technical limitations prevented analysis of the aSyn C terminus after amino acid 103. Complementary work by Ubbiali *et al.* (*68*) avoided this problem by adding an additional digestion step with an endoproteinase GlucC after a cross-linking reaction. Thus, they were able to observe C-terminal–to–core contacts and N-terminal–to–C-terminal as well. Our data and others suggest that aSyn monomer is more compact than expected for an unfolded disordered protein (*69*–*71*).

To interpret potential changes in conformation of the aSyn N terminus, we leveraged a published ensemble of aSyn(WT) monomer conformations derived from experimental restraints using paramagnetic relaxation enhancement NMR (Protein Ensemble Database, PED00024). The N terminus of aSyn from the ensemble was clustered, revealing four dominant conformations. We found that one cluster that contained 19.4% of the ensemble (cluster 1; fig. S4E) was consistent with the XL-MS data, especially N-terminal cross-links in which 12 of 15 cross-linked amino acid pairs were geometrically compatible (<30 Å; fig. S4, E and F). We observed a compact N terminus within that model, where negatively charged amino acid E13 was surrounded by positively charged lysines (fig. S4G).

Because E13K increased the local positive charge at the N terminus, we hypothesized that it might destabilize the N-terminal local structure, most likely by electrostatic repulsion of surrounding lysines. This idea was supported by NMR, which indicated changes in the chemical environment of several residues in response to the E13K substitution (amino acids 4 to 23) at a distance beyond that predicted from an unfolded peptide (*28*, *72*, *73*). The residues that exhibited chemical shift changes between aSyn(WT) and aSyn(E13K) were detected as cross-links in the aSyn(WT) XL-MS. NMR data obtained in the absence of salts suggested that E13K may also change interactions between the N and C terminus (fig. S5, F to J) (*74*), which we were not able to detect by cross-linking because of the limitations mentioned above. According to this model, E13K would be expected to enhance interactions of the N terminus with the negatively charged C terminus and decrease aggregation. However, we observed the opposite—aggregation enhancement. This suggests that interactions of the N terminus with the hydrophobic aSyn core are dominant and play a more important role in modulation of aggregation. Previous reports suggested the presence of transient N-terminal helical structure in aSyn monomer, enhanced by the N-terminal acetylation (*52*, *75*). Thus, we expect that N-terminal acetylation, as well as other PTMs, could influence aggregation propensity of aSyn monomer and its N-terminal local structure. In summary, we have concluded that aSyn monomer contains N-terminal local structure that hinders aggregation.

### More sensitive and specific biosensors

Analysis of truncated aSyn indicated the importance of key domains and suggested an important inhibitory role of the termini. The increased aggregation propensity of aSyn truncations has also been proposed by other groups (*46*–*48*). We identified residues 35 to 110 as the most aggregation-prone unit. The addition of the disease-linked mutations (E46K, H50Q, and A53T) slightly increased the seeding capacity of this truncated sequence (fig. S3A). These constructs represented more sensitive aSyn biosensors—at least fourfold moreso than full-length WT aSyn. We tested the panel of biosensors with sarkosyl-insoluble fibrils from MSA, PD, and LBD brain tissue. In agreement with data reported by Prusiner and colleagues (*8*), we observed that MSA material did not seed on biosensors containing the E46K mutation (either full length or truncated), but we found that LBD and PD fibrils seeded especially well on the truncated biosensor expressing 35 to 110 aSyn (E46K). Thus, the aSyn termini inhibited the seeding by brain-derived fibrils. These results are consistent with reported structural differences between MSA and LBD fibrils (*42*, *43*). The MSA fibrils have a completely distinct amyloid core (*42*, *43*), which probably explains their seeding profile in the biosensors. Last, we found that two biosensors were sufficient to discriminate LBD and PD versus MSA fibrils: 35 to 110 E46K and A53T. These reagents may represent useful tools for characterization of synucleinopathies.

## MATERIALS AND METHODS

All experiments in this study were performed with adherence to ethical regulations. Human brain samples were derived from individuals followed in the neurology clinics at Washington University in St. Louis and the University of Texas Southwestern Medical Center, who agreed to donate their brain tissue for research purposes. Deceased subjects are not considered human subjects for research purposes; thus, these studies were considered exempt from human subject research regulations and did not require institutional review board approval. Human materials were deidentified to ensure that no identifiable health information was available to the investigators.

### Recombinant aSyn expression and purification

We designed aSyn gene blocks with sequences of interest, which were synthesized by Integrated DNA Technologies (IDT). We used present NdeI and SpeI restriction sites to subclone these genes into ampicillin-resistant pTXB1 plasmid (NEB N6707S) by ligation. *Escherichia coli* BL21(DE3) cells were transformed with pTXB1 aSyn plasmids, plated into ampicillin plates, and incubated overnight at 37°C. One hundred milliliters of Luria broth (LB) with ampicillin (100 μg/ml) was inoculated with a single colony and grown overnight at 37°C. The culture was used for inoculation of 2 liters of LB medium with ampicillin (20 ml of the culture per 2 liters of LB medium). The bacteria were grown until OD_600_ (optical density at 600 nm) 0.5 and then induced with isopropyl-β-d-thiogalactopyranoside (IPTG) to a final concentration of 0.4 mM. The culture was grown for another 3 hours. Bacteria were harvested by centrifugation at 5000 rpm for 20 min at 4°C. Obtained pellets were resuspended in 30 to 50 ml of lysis buffer [20 mM Na-Hepes and 500 mM NaCl (pH 8.5)], frozen, and stored at −80°C until use. The resuspended pellets were quickly thawed, and the cells were lysed with a French press at 10,000 to 15,000 psi in a 5-min cycle until the liquid became transparent. The cell solution was centrifuged at 15,000*g* for 30 min at 4°C. Then, the supernatant was passed through a 22-μm cutoff filter. For further protein purification, we packed a column with 40 ml of slurry of chitin beads (Chitin Resin, NEB, S6651L). The resin was washed with 200 ml of the lysis buffer. Next, we loaded the filtered supernatant into the prepared chitin column at 0.5 to 1 ml/min, ensuring that the resin was never dry. Then, we added 50 ml of cleavage buffer [20 mM Na-Hepes, 500 mM NaCl, and 50 mM dithiothreitol (DTT) (pH 8.5)], quickly collecting 30 ml of flow-through (discarded) and incubating chitin beads with the rest of the cleavage buffer for 48 hours in darkness at 4°C. Then, we collected the flow-through containing eluted aSyn. Next, we added another 40 ml of cleavage buffer and incubated chitin beads for another 48 hours. Flow-through was combined and dialyzed twice overnight at 4°C into 5 liters of 10 mM ammonium bicarbonate using dialysis cassettes with 10-kDa protein molecular weight cutoff with a delicate mixing. After dialysis, the sample was passed through a 22-μm cutoff filter, flash-frozen, and stored at −80°C. Next, we lyophilized the protein sample and stored it at −80°C until further use. The sample was then dissolved in 100% trifluoroacetic acid (TFA) and diluted with water to 50% TFA, and resolved using a reversed-phase column (Zorbax, 300SB-C3). The protein was eluted with a gradient of increasing concentration of acetonitrile with constant 0.05% TFA. We combined collected fractions containing aSyn monomer and dialyzed them twice overnight at 4°C into 5 liters of 10 mM ammonium bicarbonate. The dialyzed sample was filtered with a 22-μm cutoff filter and flash-frozen. The sample was then lyophilized and stored at −80°C.

### aSyn fibrillization

Lyophilized WT aSyn monomer was resuspended in a buffer containing 20 mM tris, 100 mM NaCl at pH 8.0 to a concentration of 2 mg/ml (138 μM). The sample was incubated at 37°C in a Thermomixer (Eppendorf) with shaking at 1000 rpm for 7 days. The sample was stored at 4°C.

### Transmission electron microscopy

A total of 4.5 μl of sample [0.1 mg/ml of recombinant aSyn(WT) fibrils or 0.2 mg/ml of brain-derived sarkosyl-insoluble material] was applied to a glow-discharged grid (glow-discharged for 25 s; Pelco easiGlow; carbon film 300 mesh, copper; CF300-CU-50, Electron Microscopy Sciences) and incubated for 1 min at room temperature (RT). After that, the grid was blotted and quickly washed with 4.5 μl of water. Next, the grid was stained with 4.5 μl of 2% uranyl acetate for 45 s, blotted, and dried at RT. Images were acquired using a Tecnai G2 spirit transmission electron microscope (FEI, Hillsboro, OR), serial number D1067, equipped with a LaB6 source at 120 kV and a Gatan ultrascan charge-coupled device camera.

### Library preparation

We ordered 35– to 61–nucleotide (nt)–long primer pairs with degenerated codons NNS in the middle of each primer (N = G/C/A/T, S = G/C), flanked by 10 to 48 nt matching the WT nucleotide aSyn sequence (IDT). We individually cloned 139 saturated mutants for each residue and pooled plasmids with all the mutants to one tube in a mass ratio of 1:1. Cloning of each mutated residue consisted of seven steps.

#### (i) Extension PCR

Two halves of aSyn nucleotide sequence were amplified using two primer pairs (vector forward + NNS reverse; NNS forward + vector reverse). For each reaction, we added 0.5 μl of iProof polymerase (Bio-Rad, 1725301), 1 μl of 10 mM deoxynucleotide triphosphate (dNTP) [New England Biolabs (NEB), N0447L], 1 μl of 100% dimethyl sulfoxide (DMSO), 1 μl of template (2 to 20 ng, kanamycin-resistant vector containing WT aSyn sequence), 1 μl of 10 μM primer mixture (forward + reverse) at a molar ratio of 1:1, 10 μl of 5× iProof buffer, and 36 μl of nuclease-free water.

Polymerase chain reaction (PCR) program:

$$\begin{array}{c} 98{}^{\circ }C\;3\;\min\\ \begin{array}{c} 98{}^{\circ }C\;10\;s\\ 55{}^{\circ }C\;20\;s\\ 72{}^{\circ }C\;15\;s \end{array}\}\;20\;\mathrm{cycles}\\ 72{}^{\circ }C\;5\;\min\\ 4{}^{\circ }C\;\infty \end{array}$$

#### (ii) Overlap PCR

We mixed two halves of aSyn gene from the previous step to build one fragment (insert). For each reaction, we added 0.5 μl of iProof polymerase (Bio-Rad, 1725301), 1 μl of 10 mM dNTP (NEB, N0447L), 1 μl of 100% DMSO, 1 μl of template (0.5 μl + 0.5 μl of each fragment from the extension PCR), 1 μl of 10 μM primer mixture at a molar ratio of 1:1 (forward + reverse vector primers), 10 μl of 5× iProof buffer, and 36 μl of nuclease-free water.

PCR program:

$$\begin{array}{c} 98{}^{\circ }C\;3\;\min\\ \begin{array}{c} 98{}^{\circ }C\;10\;s\\ 72{}^{\circ }C\;20\;s\\ 72{}^{\circ }C\;20\;s \end{array}\}\;20\;\mathrm{cycles}\\ 72{}^{\circ }C\;5\;\min\\ 4{}^{\circ }C\;\infty \end{array}$$

#### (iii) Insert purification

Reaction mixtures after the overlap PCR, containing inserts for the following steps, were purified with a DNA purification kit (Zymo Research Genomic DNA Clean & Concentrator-10 D4011), eluting DNA with 43 μl of nuclease-free water.

#### (iv) Insert digestion

We digested purified inserts with an Esp3I restriction enzyme. For each reaction, we added 42.5 μl of purified insert from step (iii), 2.5 μl of 20 mM DTT, 5 μl of 10× Fast Digest buffer, and 1 μl of Esp3I restriction enzyme. The reactions were incubated at 37°C for 1 hour, then at 65°C for 10 min, and at 4°C until later use.

#### (v) Ligation

We ligated digested inserts with a digested target vector containing a cytomegalovirus (CMV) promoter, an mEos C-terminal tag, and ampicillin resistance. For each reaction, we added 2 μl of 10× T4 DNA ligase buffer, 50 ng of vector DNA, 9 μl of digested insert DNA from step (iv), and nuclease-free water to fill it up to 20 μl. Next, we added 1.1 μl of T4 DNA ligase, the mixture was incubated at 16°C for 18 hours and then at 65°C for 10 min, and the temperature was lowered to 4°C until further use.

#### (vi) Electroporation

We electroporated 1 μl of each ligation reaction into 19.5 μl of 10-Beta electrocompetent *E. coli* (NEB, C3020K) with a Bio-Rad *E. coli* Pulser (2.0 kV, 200 ohms, 25 μF) and 0.1-cm gap cuvettes (Bio-Rad Gene Pulser MicroPulser cuvette, 1652089). Then, we immediately added 980 μl of super optimal medium with catabolic repressor (SOC) medium and incubated the mixture at 37°C with 220 rpm shaking for 1 hour. Then, the electroporated bacteria were plated on LB agar plates with ampicillin at 100 μg/ml using glass beads and incubated overnight at 37°C.

#### (vii) Plasmid purification

We harvested all colonies from each plate by scrapping them with 10 ml of LB medium with 100 μg/ml of ampicillin. Bacteria were spun down at 4°C, 3901*g* for 10 min. Plasmids were purified from the pelleted bacteria with a Qiagen kit (QIAprep Spin Miniprep Kit, 27106), eluted with 50 μl of nuclease-free water, and stored at −20°C.

### Lentivirus production

Lentivirus was produced in HEK293T cells [available from the American Type Culture Collection (ATCC)] through a triple transfection with a packaging plasmid Gag-Pol and an envelope plasmid pVSV-g together with a lentiviral transfer vector carrying an aSyn gene variant (or a pooled aSyn library). HEK293T cells were plated at 400,000 cells per well in six-well plates and incubated at 37°C. The next day, 800 ng of Gag-pol, 200 ng of pVSV-g, and 1000 ng of lentiviral transfer vector were mixed with 7.5 μl of TransIT-293 (Mirus) and 92.5 μl of reduced serum medium (Gibco, Opti-MEM) and incubated at RT for 15 min. The cell medium was changed to 1 ml of DMEM (Dulbecco’s modified Eagle’s medium; Gibco, 11965118) with 3% fetal bovine serum (FBS; Sigma-Aldrich, F2442-500ML). The transfection mixture was added dropwise to the wells and incubated at 37°C. After 6 hours, the medium was changed to fresh 1.5 ml of DMEM with 3% FBS. After 48 hours, the medium was collected, stored at 4°C, and replaced with fresh 1.5 ml of DMEM with 3% FBS. The next day, the medium was collected, combined with the previous one, and centrifuged at 1000*g* for 5 min. Polybrene (Sigma-Aldrich Polybrene Infection TR-1002-G) and Hepes were added to the supernatant to a final concentration of 4 μg/ml and 50 nM, respectively. The mixture was passed through a 22-μm filter, aliquoted, and stored at −80°C.

### aSyn saturation mutagenesis screen

HEK293T cells (available from ATCC) were plated into five 10-cm dishes, 1.5 × 10^6^ cells per plate in 9 ml of compete cell medium [DMEM, GlutaMAX, 10% FBS, and penicillin-streptomycin (100 U/ml; Gibco)], and incubated at 37°C. The next day, 25 μl of aSyn library lentivirus diluted with 975 μl of Opti-MEM was added dropwise to each of the five dishes with HEK293T cells (resulting in a multiplicity of infection below 0.3). Cells were incubated at 37°C for 48 hours. Then, the cell medium was replaced (10 ml). aSyn fibrils were sonicated (or buffer for a negative control) at an amplitude of 65 for 5 min (1 min ON/1 min OFF) at 4°C in a water bath using a Q700 Sonicator (QSonica). Next, in one tube, we diluted 100 μl of sonicated aSyn fibrils or buffer with 650 μl of Opti-MEM. In the second tube, we mixed 50 μl of Lipofectamine (Invitrogen, Lipofectamine 2000 Transfection Reagent, 11-668-500) with 700 μl of Opti-MEM and incubated the mixture at RT for 5 min. After that, both mixtures were combined and incubated at RT for 30 min. The total of 1.5 ml of prepared treatment (with fibrils or Lipofectamine only) was added to each of three plates with HEK293T cells. The fibrils were diluted to a final concentration of 200 nM. Cells were incubated with a treatment at 37°C for 48 hours. Next, the cells were harvested by removal of cell medium, treatment with 2 ml of 0.05% trypsin (Gibco, trypsin-EDTA 0.05% and phenol red, 25300120), and incubation at 37°C for 2 min. 8 ml of complete medium was added to stop trypsinization. The cells were centrifuged at 1000 rpm for 5 min and fixed with 5 ml of 2% paraformaldehyde (PFA) in Dulbecco’s phosphate-buffered saline (DPBS; Gibco, 14-190-250) at RT for 10 min. The cells were centrifuged at 1000 rpm for 5 min, resuspended in 1 ml of DPBS, and diluted by adding another 4 ml of DPBS and placed into 6-cm dishes. The cells were treated with UV light (~405 nm) for 1 hour to convert green mEos to red. The cells were centrifuged at 1000 rpm for 5 min, resuspended in 1 ml of DPBS, passed through 50-μm filters, and diluted in 5 ml of DPBS. Moreover, we prepared fixed HEK293T cells with aSyn A53T-dsRed, aSyn A53T-mEos, and WT HEK293T cells for FACS compensation controls. After cell preparation, the cells were sorted at 4°C to DPBS into FRET-positive and FRET-negative gate using FACSAria II SORP with the four-way purity sorting method. The gating strategy was followed as previously described (*76*). Lasers and filters used: laser 488 nm excitation (Ex) 50 mW, long pass (LP) filter 600 nm, band pass (BP) filter 610/20 nm (for mEos FRET); 561 nm Ex 100 mW, LP 595, BP filter 610/20 nm (for red mEos); 488 nm Ex 50 mW, LP 505 nm, BP filter 525/50 nm (for green mEos). Collected cells were centrifuged at 4°C, 3901*g* for 10 min. Each pellet was resuspended in 500 μl of DPBS and stored at 4°C.

### Extraction of genomic DNA

We added 500 μl of lysis buffer [11 mM EDTA, 10 mM tris-HCl (pH 8.0), 100 mM NaCl, proteinase K (0.2 mg/ml; Invitrogen, AM2548), and 0.4% SDS] to each sample containing cell pellets (maximum of 1.5 × 10^6^ cells per sample) in 500 μl of DPBS. Samples were incubated overnight at 55°C with 550 rpm shaking. After that, samples were heated up at 95°C for 10 min and then cooled at 4°C. Next, we added 2 μl of ribonuclease A (5 mg/ml; Lucigen, MRNA092) to each sample, vortexed, and incubated at 37°C with 550 rpm shaking in a Thermomixer (Eppendorf) for 1 hour. Cell solutions were added to pelleted phase lock gel (PLG) tubes (QuantaBio Phase Lock Gel Light, 10847-800). We added 500 μl of buffered phenol (pH 7.9) (Invitrogen, AM9710) to each sample, gently mixed, and centrifuged at 16,000*g* at RT for 5 min. Next, we added 500 μl of phenol-chloroform-isoamylalcohol 25:24:1 (v/v) (Invitrogen, 15593031), gently mixed, and centrifuged at 16,000*g* at RT for 5 min. Upper aqueous phases were transferred into centrifuged PLG tubes, and then 500 μl of chloroform (Sigma-Aldrich, C2432) was added to each tube, gently mixed, and centrifuged at 16,000*g* at RT for 5 min. Next, upper aqueous phases were transferred into 2-ml tubes with 1.3 μl of glycoblue (10 mg/ml; GlycoBlue Coprecipitant, Invitrogen, AM9515) and gently mixed. We added 1 ml of 100% ethanol to each sample, gently mixed, and incubated at −80°C for 1 hour. After that, samples were centrifuged at 15,000*g* at 4°C for 15 min. Pelleted DNA was washed with 1 ml of 75% ethanol (stored at −20°C) and centrifuged at 15,000*g* at 4°C for 15 min. The supernatants were aspirated, and DNA pellets were dried for several minutes. We added 50 μl of nuclease-free water and let DNA solubilize at 4°C overnight and left it at 4°C until further use.

### DNA amplification

The extracted genomic DNA from each sample for FRET-positive and FRET-negative populations was used in two rounds of PCRs, in which barcodes and adapters were added to the ends of the aSyn gene required for further deep sequencing. In the case of FRET-positive cells, all extracted DNA was used in the first PCR (Touchdown PCR1); in the case of FRET-negative cells, we used 1.6 μg of genomic DNA in the first PCR. All used primers were synthesized by IDT.

#### Touchdown PCR 1

For each reaction, we mixed 0.5 μl of iProof polymerase (Bio-Rad, 1725301), 1 μl of 10 mM dNTP (NEB, N0447L), 1 μl of 100% DMSO, template (genomic DNA), 1 μl of 10 μM mixed primers (forward and reverse) at a molar ratio of 1:1, 10 μl of 5× iProof buffer, and nuclease-free water with a total volume of 50 μl. The polymerase was added by the end of the first step of PCR (“hot start” at 98°C, 3-min step).

PCR program:

$$\begin{array}{c} 98{}^{\circ }C\;3\;\min\;\mathrm{polymerase}\;\mathrm{added}\\ \begin{array}{c} 98{}^{\circ }C\;30\;s\\ 69{}^{\circ }C\;45\;s^{*} \end{array}\}\;10\;\mathrm{cycles}\\ 72{}^{\circ }C\;1\;\min \end{array}$$

*temperature decreased by 1°C per cycle.

$$\begin{array}{c} 98{}^{\circ }C\;30\;s\\ \begin{array}{c} 59{}^{\circ }C\;45\;s\\ 72{}^{\circ }C\;1\;\min\\ 72{}^{\circ }C\;5\;\min \end{array}\}\;18\;\mathrm{cycles}\\ 4{}^{\circ }C\;\infty \end{array}$$

Used primers:

Forward:

TGACTGGAGTTCAGACGTGtgctcttccgatctNNNNN(N)_1–4_GCTAGCGCCGCCACCATG

Reverse:

CACTCTTTCCCTACACGACgctcttccgatctNNNNN(N)_1–4_GCAGCGGAGCCAGCAGAAC

#### Gel purification

The PCR product was run on a 3% agarose gel (Invitrogen UltraPure Agarose-1000, 16550100) at 80 V for 6 hours. The 532– to 538–base pair (bp) PCR product was cut out from the gel and purified with a purification kit (Promega Wizard SV Gel and PCR Clean-Up System, A9281). The eluted product was purified with a DNA purification kit (Zymo Research Genomic DNA Clean and Concentrator-10 D4011), eluting DNA with 10.5 μl of nuclease-free water.

#### PCR 2

For each reaction, we mixed 0.5 μl of iProof polymerase (Bio-Rad, 1725301), 1 μl of 10 mM dNTP (NEB, N0447L), 1 μl of 100% DMSO, 6 ng of template (purified product from step 2), 1 μl of 10 μM mixed primers (forward and reverse) at a molar ratio of 1:1, 10 μl of 5× iProof buffer, and nuclease-free water with a total volume of 50 μl. The polymerase was added by the end of first step of PCR (hot start at 98°C, 3-min step).

PCR program:

$$\begin{array}{c} 98{}^{\circ }C\;3\;\min\;\mathrm{polymerase}\;\mathrm{added}\\ \begin{array}{c} 98{}^{\circ }C\;10\;s\\ 67{}^{\circ }C\;20\;s\\ 72{}^{\circ }C\;20\;s \end{array}\}\;16\;\mathrm{cycles}\\ 72{}^{\circ }C\;5\;\min\\ 4{}^{\circ }C\;\infty \end{array}$$

We used combination of total of eight following primers for each sample:

Forward:

CAAGCAGAAGACGGCATACGAGATcgagtaatGTGACTGGAGTTCAGACGTG

CAAGCAGAAGACGGCATACGAGATtctccggaGTGACTGGAGTTCAGACGTG

CAAGCAGAAGACGGCATACGAGATaatgagcgGTGACTGGAGTTCAGACGTG

CAAGCAGAAGACGGCATACGAGATggaatctcGTGACTGGAGTTCAGACGTG

Reverse:

AATGATACGGCGACCACCGAGATCTACACtatagcctACACTCTTTCCCTACACGAC

AATGATACGGCGACCACCGAGATCTACACatagaggcACACTCTTTCCCTACACGAC

AATGATACGGCGACCACCGAGATCTACACcctatcctACACTCTTTCCCTACACGAC

AATGATACGGCGACCACCGAGATCTACACggctctgaACACTCTTTCCCTACACGAC

#### Gel purification

The PCR product was run on a 3% agarose gel (Invitrogen UltraPure Agarose-1000 16550100) at 80 V for 7 hours. The 603- to 609-bp PCR product was cut out from the gel and purified with a purification kit (Promega Wizard SV Gel and PCR Clean-Up System A9281). The eluted product was purified with a DNA purification kit (Zymo Research Genomic DNA Clean & Concentrator-10 D4011), eluting DNA with 25 μl of nuclease-free water.

### Sequencing

Deep sequencing was performed at the UTSW Next Generation Sequencing Core, using Illumina MiSeq v2 (500-cycle) Reagent Kit​ MS-102-2003. The 4 nM DNA sample was denatured with 0.2 N NaOH at 1:1 (v/v), diluted to 8 pM, and spiked with a denatured PhiX control library.

### Processing of deep sequencing data

Paired-end reads were joined using NGmerge (*77*) with default parameters. To count the number of alleles, we generated a database of expected gene sequences by substituting each codon of the WT aSyn gene with the codons encoded by NNS, where N corresponds to A, C, G, or T, and S corresponds to G or C. For each sequence in this database, we counted the number of perfect occurrences in the sequencing output. Counts corresponding to sequences with synonymous mutations were combined, and an RE score was calculated for each protein variant by comparing the observed frequency of each protein sequence in the selected population to the input frequency, according to the equation

$$\mathrm{RE}=\log\left(\frac{f_{i}^{\mathrm{sel}}}{f_{i}^{\mathrm{inlib}}}\right)-\log\left(\frac{f_{\mathrm{WT}}^{\mathrm{sel}}}{f_{\mathrm{WT}}^{\mathrm{inlib}}}\right)$$

where *f_i_^y^* is the frequency of protein *i* in sample *y*, and the reference sequence (WT) is the WT aSyn protein sequence. This RE value indicates the fold change of abundance for each protein variant relative to WT.

### Mutagenesis analysis

All analysis described in this section was performed in MATLAB (R2019a).

### Inhibitory residue classification

To identify which residues had strong inhibitory signal, we took the median signal across all mutations (missing data were treated as NaN, and data for stop codons were excluded) for all residues and applied the Otsu threshold to these data. All residues with a median value equal to or less than the threshold (−1.2152) were classified as having strong inhibitory signal for aggregation.

### t-SNE analysis

To evaluate the relationship of different mutations across all residues, we applied t-SNE dimensionality reduction to the mutagenesis data. Each residue’s 20 mutations (missing data were given a zero value) were used as input, t-SNE was performed with a perplexity value of 30, and Euclidean distance was used as the distance metric.

### Generation of aSyn biosensors

We designed aSyn gene blocks either WT or containing mutations of interest, which were synthesized by IDT. We subcloned these genes by ligation into ampicillin-resistant vectors with a CMV promoter in variants containing CFP or YFP C-terminal tags. All aSyn biosensor variants were engineered through a transduction of HEK293T cells (available from ATCC) with a lentivirus carrying an aSyn gene variant under a CMV promoter.

HEK293T cells were plated at 200,000 cells per well in 2 ml of complete DMEM in six-well plates. Two hundred fifty microliters of the lentivirus with a CFP variant and 250 μl with a YFP variant were added dropwise to the well. The cells were incubated at 37°C and maintained until the full expression of CFP and YFP was obtained. Then, the cells with aSyn variants were sorted using FACS into double positive for CFP and YFP single cells or polyclonal cell lines [lasers and filters: laser 405 nm Ex 100 mW, LP 505 nm, BP filter 525/50 nm (for FRET); 488 nm Ex 50 mW, LP 505 nm, BP filter 525/50 nm (for YFP); 405 nm Ex 100 mW, LP none, BP filter 450/50 nm (for CFP)]. Cells were grown, tested, and stored in liquid nitrogen.

### aSyn FRET seeding assay

Biosensor cells were plated at 25,000 cells per well in 130 μl of complete DMEM in a 96-well plate and incubated at 37°C. After 24 hours, recombinant full-length WT aSyn fibrils were diluted in a buffer [20 mM tris (pH 8.0) and 100 mM chloride sodium] and sonicated at an amplitude of 65 for 5 min (1 min ON and 1 min OFF) using a Q700 Sonicator (QSonica). 1 μl of sonicated fibrils (or buffer for a negative control) were diluted with 9 μl of Opti-MEM. 0.6 μl of Lipofectamine 2000 was mixed with 9.4 μl of Opti-MEM and incubated for 5 min at RT. The diluted fibrils were mixed with Lipofectamine and incubated for 30 min at RT. Twenty microliters was added dropwise to each well. Each treatment was performed in triplicate. Cells were incubated at 37°C for 48 hours. The medium was removed and 50 μl of 0.05% trypsin was added to each well and incubated for 2 min at 37°C. 100 μl of 2% PFA in DPBS was added to each well, mixed, and incubated for 10 min at RT. Plates were centrifuged at 1000 rpm for 5 min. The supernatant was removed, and cells were resuspended in 130 μl of DPBS for analysis by flow cytometry. FRET-positive cells were quantified by flow cytometry using the gating strategy described as previously (*76*) [lasers and filters: laser 405 nm Ex 50 mW, LP 475 nm, BP filter 525/50 nm (for FRET); 488 nm Ex 50 mW, LP 505 nm, BP filter 530/30 nm (for YFP); 405 nm Ex 50 mW, LP none, BP filter 450/50 nm (for CFP)].

### aSyn fibril extraction from human brain tissue

aSyn fibrils were extracted from tissue following the protocol published by Schweighauser *et al.* (*42*). Frozen human brain tissue (0.5 g) was homogenized in 10 ml of extraction buffer [10 mM tris-HCl (pH 7.5), 0.8 M NaCl, 10% sucrose, and 1 mM EGTA]. Sarkosyl was added to 2% (w/v), and the homogenates were incubated at 37°C with 1000 rpm shaking in a Thermomixer (Eppendorf). The homogenates were spun down for 10 min at 10,000*g*, 20°C. Supernatants were centrifuged for 20 min at 100,000*g*, 20°C. Pellets were resuspended in 250 μl of the extraction buffer per 0.5 g of tissue and spun down for 5 min at 3000*g*, 20°C. The supernatant was diluted with the following buffer [one part supernatant, two parts of the buffer: 50 mM tris-HCl (pH 7.5), 0.15 M NaCl, 10% sucrose, and 0.2% sarkosyl] and centrifuged for 30 min at 166,000*g*, 20°C. The supernatant was discarded, and the pellet was washed twice with PBS, followed by centrifugation. Pellets were resuspended in PBS. In case of low solubility, the pellets were sonicated at 4°C in a water bath up to 20 min at an amplitude of 65 (1-min ON and 1-min OFF) using a Q700 Sonicator (QSonica). Protein concentrations were measured with micro-BCA protein assay kit (Thermo Fisher Scientific, 23235). Samples were flash-frozen and stored at −80°C.

### Western blot

Fifteen micrograms of brain-derived sarkosyl-insoluble material was mixed with sample buffer (Bio-Rad, 1610747) supplemented with β-mercaptoethanol and boiled for 6 min. Samples and a protein MW ladder (Bio-Rad, 1610373) were loaded onto a NuPage bis-tris polyacrylamide (4 to 12% gradient) gel (Invitrogen, NP0323BOX). Electrophoresis was performed in MES-SDS buffer (Invitrogen, NP0002) for 1 hour 10 min at 150 V. Protein was transferred from the gel into a polyvinylidene difluoride (PVDF) membrane (Immobilon-P PVDF Membrane, MilliporeSigma IPVH00010) at 20 V for 50 min using a Bio-Rad Trans-blot semi-dry transfer apparatus. The membrane was blocked in 5% nonfat milk (Bio-Rad, 1706404) in TBS (tris-buffered saline) with 0.1% Tween-20 (TBST) for 1 hour at RT with agitation. Then, the membrane was incubated with an anti-aSyn rabbit antibody targeting C-terminal residues 118 to 123 (Abcam, MJFR1, ab138501) diluted 1:1000 in TBST with 5% milk overnight at 4°C with agitation. Then, the membrane was washed with TBST and incubated with a secondary antibody against rabbit immunoglobulin G (IgG) [Amersham ECL rabbit IgG, horseradish peroxidase–linked F(ab′)₂ fragment (from donkey); Cytiva, NA9340-1ML] diluted 1:5000 in TBST with 5% milk for 1 hour at RT with agitation. The membrane was washed with TBST and then incubated for 2 min with a chemiluminescence detection reagent (Cytiva, RPN2232).

### Cross-linking with MS

Lyophilized aSyn was resuspended in buffer containing 20 mM Na-Hepes and 100 mM NaCl (pH 8.0) and brought to 0.4 mg/ml. Thirty microliters of DMTMM (120 mg/ml) was added to 270 μl of protein solution and incubated at 37°C with 750 rpm shaking in a Thermomixer (Eppendorf) for 10 min. The reaction was quenched by adding 60 μl of 1 M ammonium bicarbonate and incubated at 37°C with 750 rpm shaking in a Thermomixer for 30 min. Next, the samples were flash-frozen and stored at −80°C.

We added Laemmli buffer to samples and boiled at 100°C for 5 min. The samples were resolved on a 1.5-mm bis-tris polyacrylamide (4 to 12% gradient) gel (Invitrogen, NuPage NP0336BOX) at 150 V for 1 hour and 10 min and stained with Coomassie G-250 (Invitrogen, SimplyBlue SafeStain, LC6065) overnight at RT with mild rotatory shaking. The next day, gels were washed with water for 2 hours at RT with mild rotatory shaking. Bands with monomeric aSyn (~14 kDa) were cut out and divided into small pieces and transferred into 1.5-ml tubes. Gel pieces were washed twice with 200 μl of water for 10 min at RT with 600 rpm shaking in a Thermomixer (Eppendorf). Gel pieces were sonicated six times in acetonitrile/50 mM ammonium bicarbonate in a ratio of 2:3 (v/v) at 65 A, 5 min each cycle (1-min ON, 1-min OFF) at 4°C in a water bath, until gel pieces became transparent. Gel pieces were then washed with 200 μl of 100% acetonitrile for 5 min at RT with 600 rpm shaking in a Thermomixer. Acetonitrile was removed, and residual liquid was vacuumed for 5 min in a freeze-dryer device (Labconco). Then, we added 150 μl of 10 mM DTT in 25 mM ammonium bicarbonate, and samples were incubated for 1 hour at 56°C with 600 rpm shaking in a Thermomixer. The tubes were cooled down, and solution was switched to 150 μl of 55 mM iodoacetamide in 25 mM ammonium bicarbonate. Samples were incubated for 45 min at RT in darkness. Next, gel pieces were washed with 200 μl of 25 mM ammonium bicarbonate, then 200 μl of 50% acetonitrile, and last 200 μl of 100% acetonitrile. After removing the last solution, samples were vacuumed for 5 min. We added 200 μl of 50 mM ammonium bicarbonate to each tube with gel pieces and 1.5 μl of MS-grade trypsin (1 μg/μl; NEB, P8101S) in water. The samples were incubated overnight (15 to 17 hours) at 37°C with 600 rpm shaking in a Thermomixer. The next day, the reaction was stopped by adding 0.6 μl of formic acid to each tube (until pH dropped to ~3). The samples were flash-frozen and stored at −80°C. Later, the samples were lyophilized and stored at −80°C.

The lyophilized samples were reconstituted in water/acetonitrile/formic acid (95:5:0.1, v/v/v) to a final concentration of ~ 0.5 μg/μl. In total, 2 μl each was injected for five technical replicates for LC-MS/MS analyses [at the UTSW Proteomics Core Facility as described previously (*63*, *78*)] on an Eksigent 1D-NanoLC-Ultra HPLC system coupled to a Thermo Orbitrap Fusion Tribrid system. Peptides were separated on self-packed New Objective PicoFrit columns (11 cm × 0.075 mm inner diameter) containing Magic C18 material (Michrom, 3-μm particle size, 200-Å pore size) at a flow rate of 300 nl/min using the following gradient: 0 to 5 min = 5% B, 5 to 95 min = 5 to 35% B, 95 to 97 min = 35 to 95% B, and 97 to 107 min = 95% B, where A = (water/acetonitrile/formic acid, 97:3:0.1) and B = (acetonitrile/water/formic acid, 97:3:0.1). The mass spectrometer was operated in data-dependent mode by selecting five of the most abundant precursor ions [mass/charge ratio (*m*/*z*) 350 to 1600, charge state 3+ and above] from a preview scan and subjecting them to collision-induced dissociation (normalized collision energy = 35%, 30-ms activation). Fragment ions were detected at low resolution in the linear ion trap. Dynamic exclusion was enabled (repeat count 1, exclusion duration 30 s).

### Processing of MS data

Thermo.raw files were converted to the open.mzXML format using msconvert (proteowizard.sourceforge.net) and analyzed using an in-house version of xQuest50. Search parameters were set based on the cross-link reagent used. DMTMM zero-length cross-linking was searched against FASTA database containing aSyn (UniProt ID P37840) or with a mutant substitution. xQuest settings for DMTMM include maximum number of missed cleavages = 2, peptide length = 5 to 50 residues, fixed modifications = carbamidomethyl-Cys (mass shift = 57.02146 Da), mass shift of cross-linker = −18.010595 Da, no monolink mass specified, MS1 tolerance = 15 parts per million (ppm), and MS2 tolerance = 0.2 Da for common ions and 0.3 Da for cross-link ions, search in enumeration mode. Post-search manual validation and filtering were performed using the following criteria: xQuest score > 30, mass error between −2.2 and +3.8 ppm, % of total ion chromatogram (%TIC) > 10, and a minimum peptide length of six amino acids. In addition, at least four assigned fragment ions (or at least three contiguous fragments) were required on each of the two peptides in a cross-link. FDRs for the identified cross-links were estimated using xprophet60. For each experiment, five replicate datasets were compared and only cross-links present in five of five datasets were used to generate a consensus dataset.

### Expression and purification of ^15^N-labeled recombinant aSyn

We designed aSyn gene blocks either WT or containing mutations of interest, which were synthesized by IDT. We used present NdeI and SpeI restriction sites to subclone these genes into ampicillin-resistant pTXB1 plasmid (NEB, N6707S) by ligation. *E. coli* BL21(DE3) cells were transformed with pTXB1 aSyn plasmids, plated into ampicillin plates, and incubated overnight at 37°C. Fifty microliters of LB with ampicillin (100 μg/ml) was inoculated with a single colony and grown overnight at 37°C. The overnight culture was centrifuged at RT, 4000*g* for 20 min. The pellet was resuspended in 50 ml of ^15^N medium with ampicillin (100 μg/ml). The ^15^N medium contained 47.9 mM Na_2_HPO_4_, 22 mM KH_2_PO_4_, 8.56 mM NaCl, 18.4 mM ^15^N NH_4_Cl, 0.1 mM CaCl_2_, 2 mM MgSO_4_, 0.05% thiamine, and 0.769% glucose. Twenty microliters of the culture was used for inoculation of 1 liter of ^15^N medium with ampicillin. Bacteria were grown to OD_600_ of 0.8 and then induced with IPTG at the final concentration of 0.4 mM. The culture was grown for another 3 hours. Bacteria were harvested by centrifugation at 5000 rpm for 20 min at 4°C. Pellets were resuspended in 30 to 50 ml of lysis buffer [20 mM Na-Hepes and 500 mM NaCl (pH 8.5)], frozen, and stored at −80°C. The ^15^N-labeled aSyn was purified following the regular protocol described above for the unlabeled protein.

### NMR spectroscopy

The ^1^H-^15^N HSQC spectra were recorded at 15°C on an Agilent DD2 spectrometer at 800 MHz using a cold probe. The samples contained 30 μM ^15^N-labeled monomeric aSyn (WT or E13K mutant) in either 20 mM Hepes (pH 7.4), 100 mM NaCl, 10% D_2_O or 20 mM sodium phosphate (pH 6.0), 10% D_2_O. Total acquisition time was 2 hours for each sample. NMR data were processed with NMRPipe (*79*) and analyzed with NMRView (*80*). The chemical shift assignments were completed on the basis of data published by Maltsev *et al*. (*52*). The following formulas were used for calculating chemical shift changes (Δδ) and chemical shift perturbations (CSP)

$$\text{Δ}\delta =\delta_{\mathrm{WT}}-\delta_{E13K}$$

$$\mathrm{CSP}=\sqrt{{\left({\text{Δ}\delta }^{1}H^{N}\right)}^{2}+{\left(\frac{{\text{Δ}\delta }^{15}N}{5}\right)}^{2}}$$

## Supplementary Materials

### This PDF file includes:

Figs. S1 to S5 Tables S1 to S3 References
