# Supplementary material for: Saturation mutagenesis of α-synuclein reveals monomer fold that modulates aggregation
Source: Sci Adv. 2023 Oct 27;9(43):eadh3457. doi: 10.1126/sciadv.adh3457 (PMC10610913; doi:10.1126/sciadv.adh3457)
Supplement: Supplementary file 1 — Figs. S1 to S5 Tables S1 to S3 References [file sciadv.adh3457_sm.pdf]

Science Advances
AAAS

Supplementary Materials for

Saturation mutagenesis of α-synuclein reveals monomer fold that modulates aggregation

Julita Chlebowicz *et al.*

Corresponding author: Marc I. Diamond, marc.diamond@utsouthwestern.edu

Sci. Adv. **9**, eadh3457 (2023)
DOI: 10.1126/sciadv.adh3457

This PDF file includes:

Figs. S1 to S5
Tables S1 to S3
References

Treatment	Amount of collected cells	
	**FRET-positive**	**FRET-negative**
No treatment	489	68,059,103
Vehicle (lipofectamine)	1,799	54,077,271
200 nM fibrils	21,172	82,318,735

Table S1. Amount of FRET-positive and -negative cells collected by FACS for each sample with or without a treatment.

Fluorophore	Excitation λ (nm)	Emission λ (nm)	Molecular weight (kDa)
ECFP (*81*)	434	477	26.8
EYFP (*82*)	513	527	26.9
mEos3.2 green (*83*)	507	516	25.6
mEos3.2 red	572	580	25.6

Table S2. Properties of fluorophores used in the study.

Category	Healthy control	MSA	PD	LBD
**Age**	87	63	80	75
**Sex**	Female	Male	Male	Male
**Clinical diagnosis**	-	Ataxia and autonomic dysfunction beginning 11 years prior to death; dx of multiple system atrophy 5 years before death	PD; clinical history of dementia	PD with dementia
**Braak stage**	-	II	III	II
**Duration**	-	11 years	15 years	17 years
**Histopathology**	No Lewy bodies, no plaques; Neurofibrillary tangles, mesial temporal lobe; Alzheimer type II astrocytisis, putamen	Multiple system atrophy with both olivopontocere-bellar and striatonigral involvement; Braak NFT stage II; CERAD plaque score 0; Thal amyloid stage 0	Pigmented neuron loss; Lewy bodies in substantia nigra; microscopic changes of Alzheimer disease, insufficient for diagnosis; sparse plaques	Lewy body disease, limbic stage; Braak NFT stage II; CERAD plaque score 0; Thal amyloid stage 0
**Brain region used for seeding**	Middle temporal gyrus	White matter	Temporal lobe (cortex)	Limbic (cingulate)

Table S3. Characteristics of patients involved in the study.

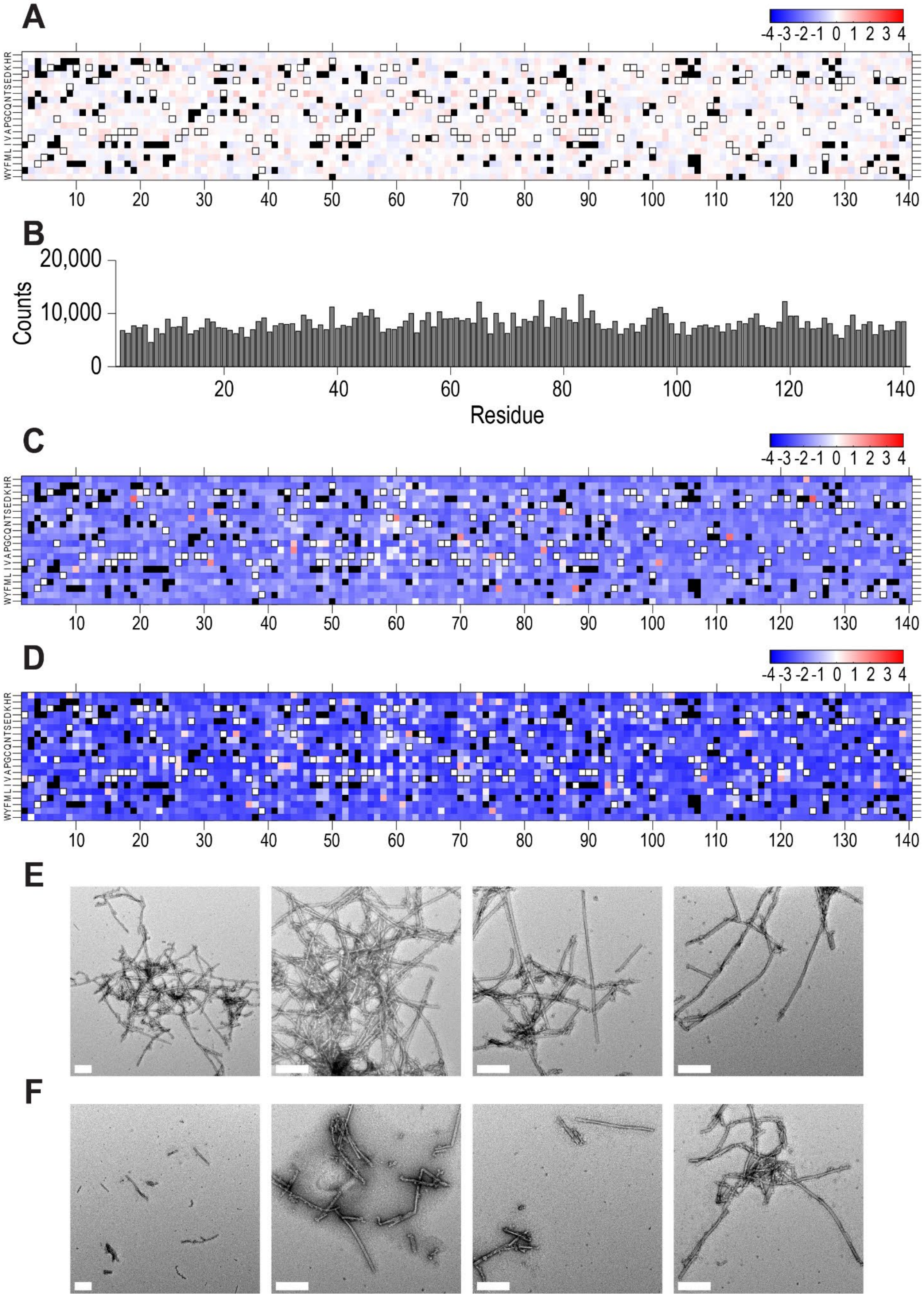
A
B
C
D
E
F
-4 -3 -2 -1 0 1 2 3 4
WYFMLIVAPGCQNTSEDKHR
Counts
20,000
10,000
0
Residue
10 20 30 40 50 60 70 80 90 100 110 120 130 140

Fig. S1. Sequencing reveals no mutant depletion nor spontaneous aggregation. (**A**) Relative enrichment (RE) of each point aSyn mutant in the library expressed in HEK293T cells. The horizontal axis shows aSyn residues 2-140; the left vertical axis indicates missense amino acid substitution in the following order: R, H, K, D, E, S, T, N, Q, C, G, P, A, V, I, L, M, F, Y, W. The scale bar indicates the log10 enrichment. White pixels with a black frame represent WT aSyn residues. Positive RE (red color scale) represents mutants with a higher representation vs. WT. Negative RE (blue color scale) represents mutants with a lower representation vs. WT. White pixels without a frame represent mutants similar to WT. Black pixels represent mutants not detected sufficiently for an accurate count and thus excluded from further analysis. (**B**) Counts for each mutated residue obtained from deep sequencing of the saturation library expressed in HEK293T cells. (**C**) Relative enrichment (RE) of each point aSyn mutant in the FRET positive population of cells with no treatment or (**D**) treated with vehicle only (Lipofectamine2000). The axes and scale are the same as in A. (**E**) TEM of aSyn(WT) recombinant fibrils before sonication. (**F**) TEM of aSyn(WT) recombinant fibrils after 5 minutes of sonication. Scale bar = 200 nm.

A

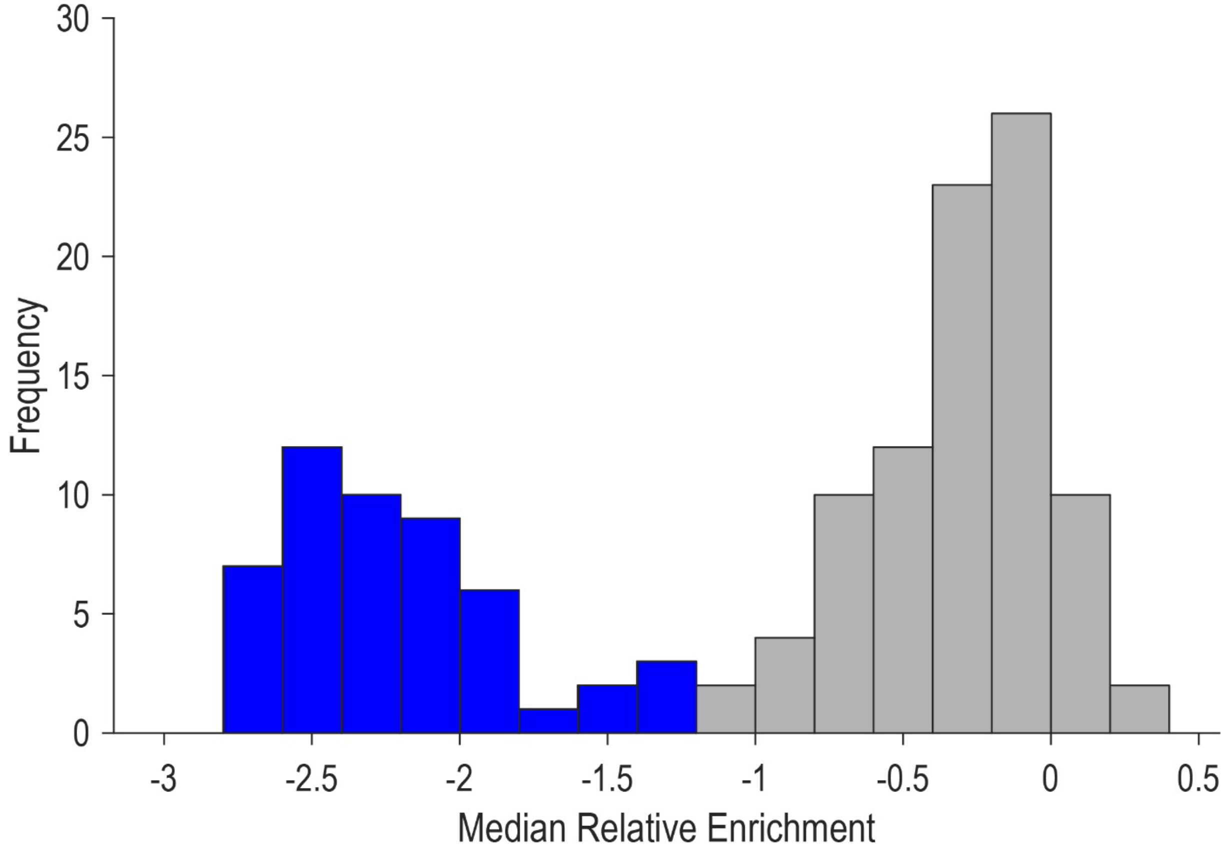

B

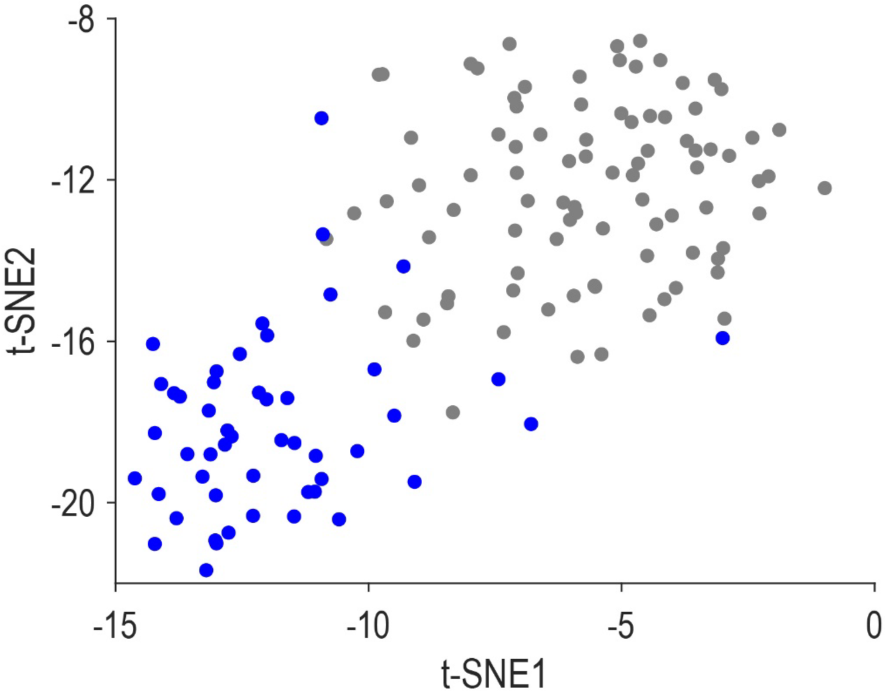

-8
-12
-16
-20
t-SNE2
-15
-10
-5
0
t-SNE1

C

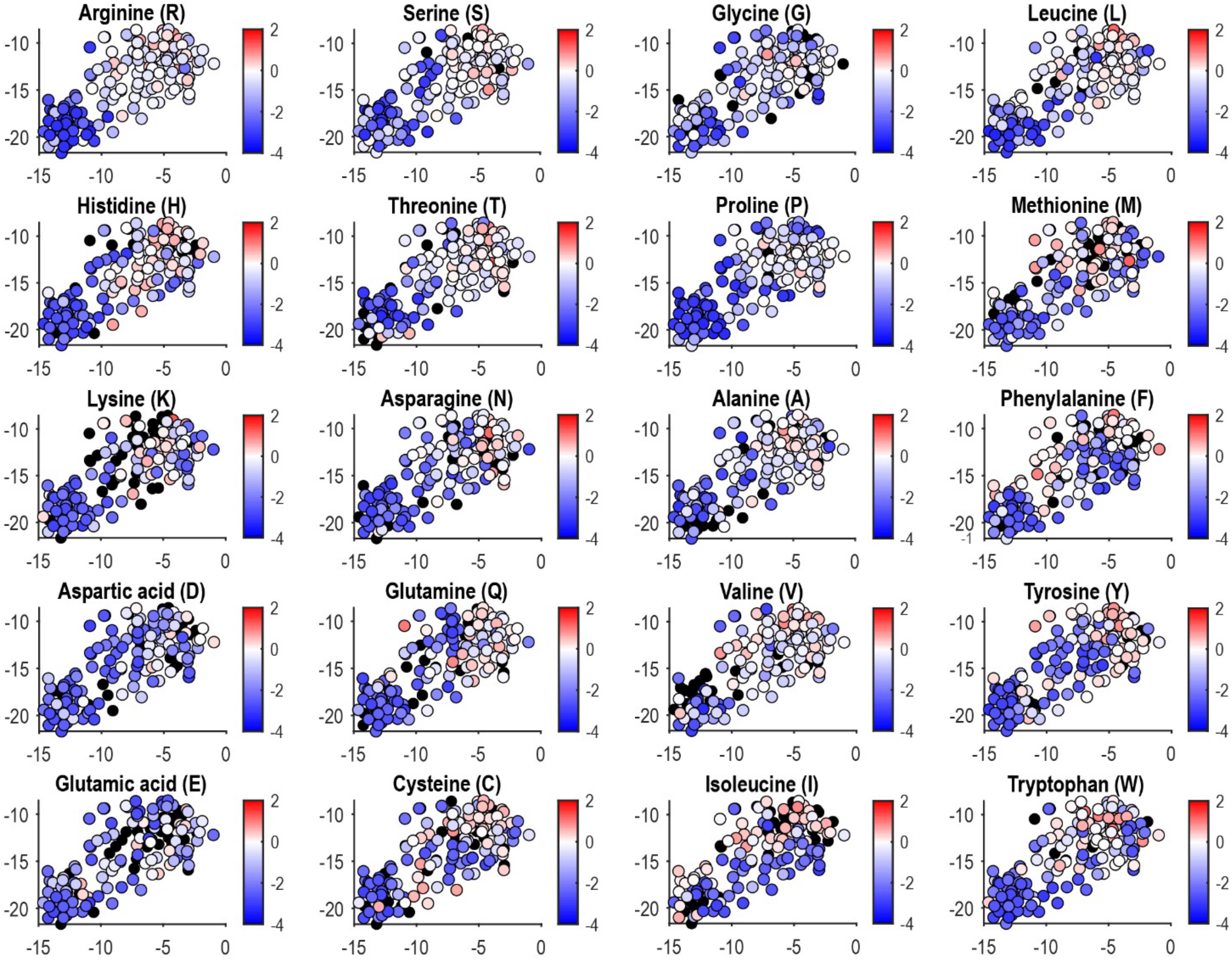

Arginine (R)
Serine (S)
Glycine (G)
Leucine (L)
Histidine (H)
Threonine (T)
Proline (P)
Methionine (M)
Lysine (K)
Asparagine (N)
Alanine (A)
Phenylalanine (F)
Aspartic acid (D)
Glutamine (Q)
Valine (V)
Tyrosine (Y)
Glutamic acid (E)
Cysteine (C)
Isoleucine (I)
Tryptophan (W)

D

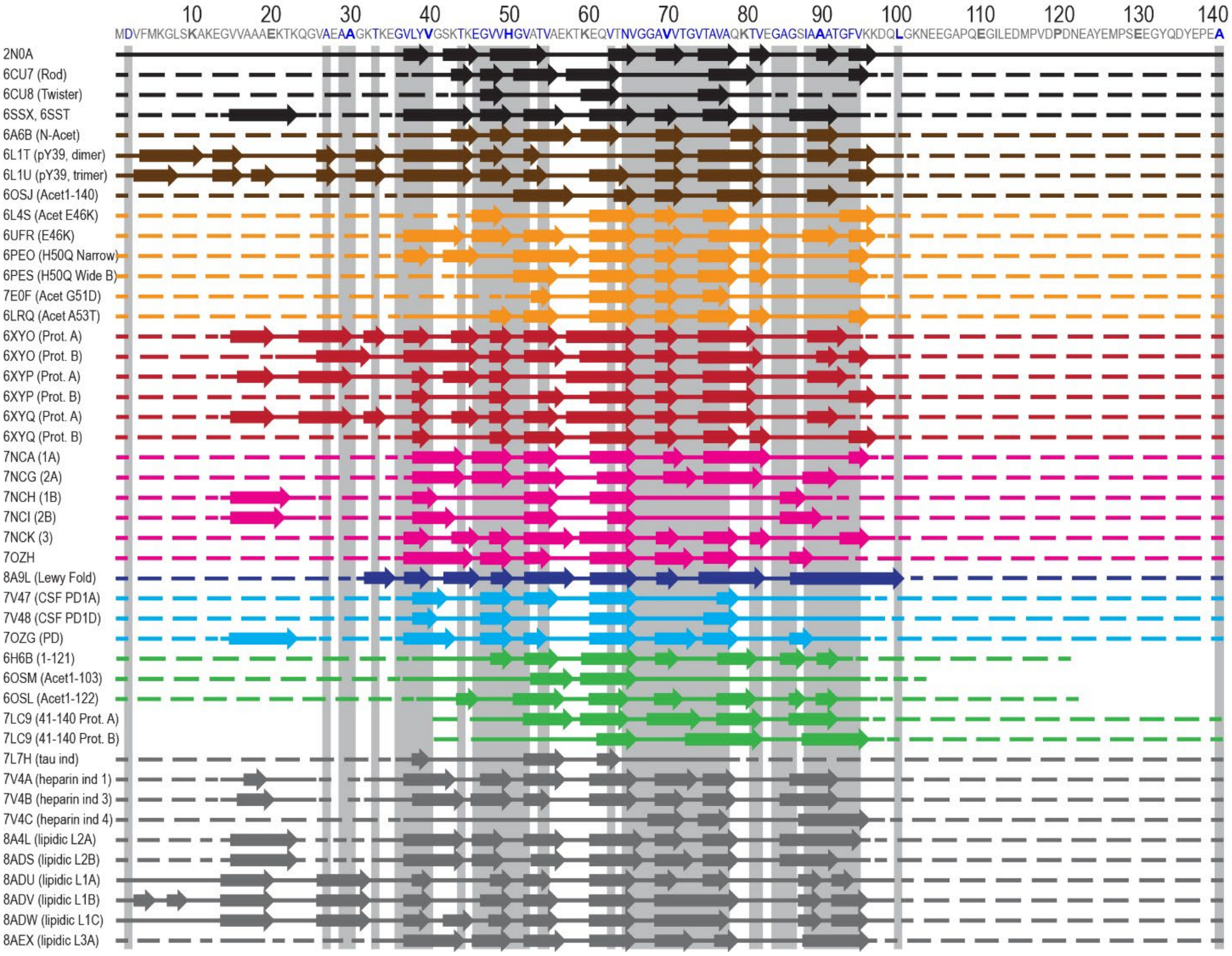

Fig. S2. Inhibitory residue classification and t-SNE reveals amino acid substitutions which act as "aggregation gatekeepers". (**A**) Histogram representing the frequency distribution of median RE within the mutagenesis data (missing data and data for stop codons were excluded). Residues with median RE equal or lower than calculated Otsu threshold (-1.2152) were marked as blue bars and identified as aSyn domains promoting seeded aggregation. (**B**) A t-SNE clustering plot with arbitrary units shows clustered mutagenesis data. Data points represent aSyn residues (2-140). Residues with relative enrichment equal or lower than calculated Otsu threshold (-1.2152) were marked as blue and identified as aSyn domains driving its aggregation. (**C**) t-SNE plots generated for each amino acid used for a substitution. Data points are colored by the relative enrichment as in the Fig. 3A. The scale bar indicates the $\log_{10}$ enrichment. Positive RE (red color scale) represents mutants over-represented vs. WT, presumably with higher aggregation propensity. Negative RE (blue color scale) represents mutants under-represented vs. WT, presumably with lower seeding propensity. White data points represent mutants similar to WT. Black data points represent mutants not detected sufficiently for an accurate count and thus excluded from further analysis. The graphs are ordered based on amino acid properties. Arginine has a unique aggregation disruptive effect in the core domains. (**D**) Comparison of secondary structures across all available ssNMR and cryo-EM aSyn datasets. Arrows indicate localization of β-sheets, a continuous line indicates regions with a core structure, a dashed line indicates aSyn "fuzzy coat." Black: structures of recombinant aSyn(WT) fibrils (*34, 35, 84*); brown: structures

of recombinant aSyn(WT) fibrils containing post-translational modifications (*36, 37*); yellow: structures of recombinant aSyn containing disease-linked mutations (*38-41, 85*); red: aSyn structures derived from MSA brain (*42*); pink: recombinant amplified MSA fibrils (*62*); dark blue: aSyn structure derived from LBD or PD brain (*43*); light blue: recombinant amplified LBD or PD fibrils (*86*); green: truncated recombinant aSyn fibrils (*44-46*); grey: recombinant aSyn fibrils induced by tau, heparin or lipids (*87*). PDB ID for each structure is indicated on left. Grey horizontal stripes indicate localization of aSyn residues driving its aggregation. β-sheets were assessed using a dss command in PyMOL.

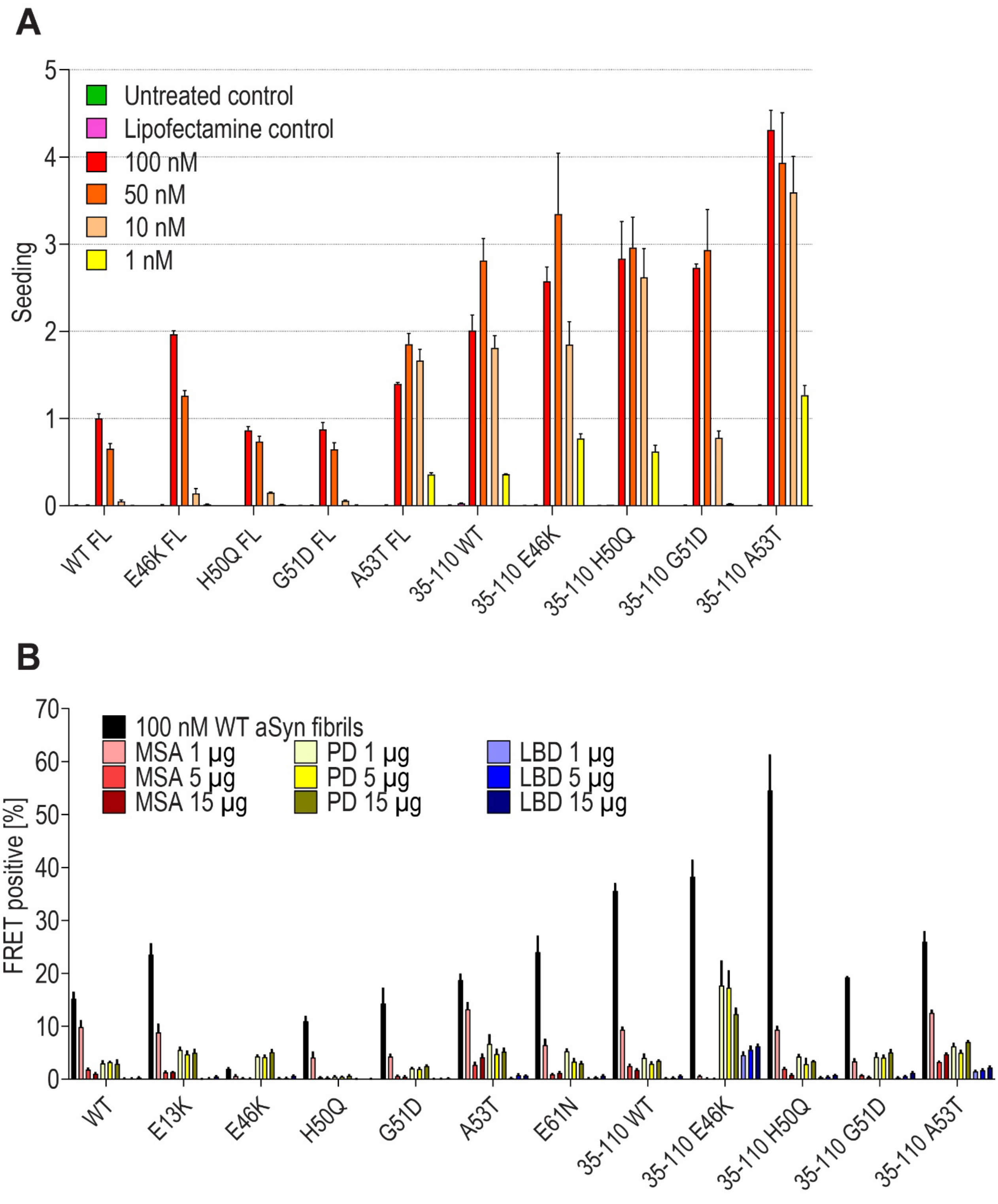
A
Untreated control
Lipofectamine control
100 nM
50 nM
10 nM
1 nM
Seeding
0
1
2
3
4
5
WT FL
E46K FL
H50Q FL
G51D FL
A53T FL
35-110 WT
35-110 E46K
35-110 H50Q
35-110 G51D
35-110 A53T
B
100 nM WT aSyn fibrils
MSA 1 µg
MSA 5 µg
MSA 15 µg
PD 1 µg
PD 5 µg
PD 15 µg
LBD 1 µg
LBD 5 µg
LBD 15 µg
FRET positive [%]
0
10
20
30
40
50
60
70
WT
E13K
E46K
H50Q
G51D
A53T
E61N
35-110 WT
35-110 E46K
35-110 H50Q
35-110 G51D
35-110 A53T

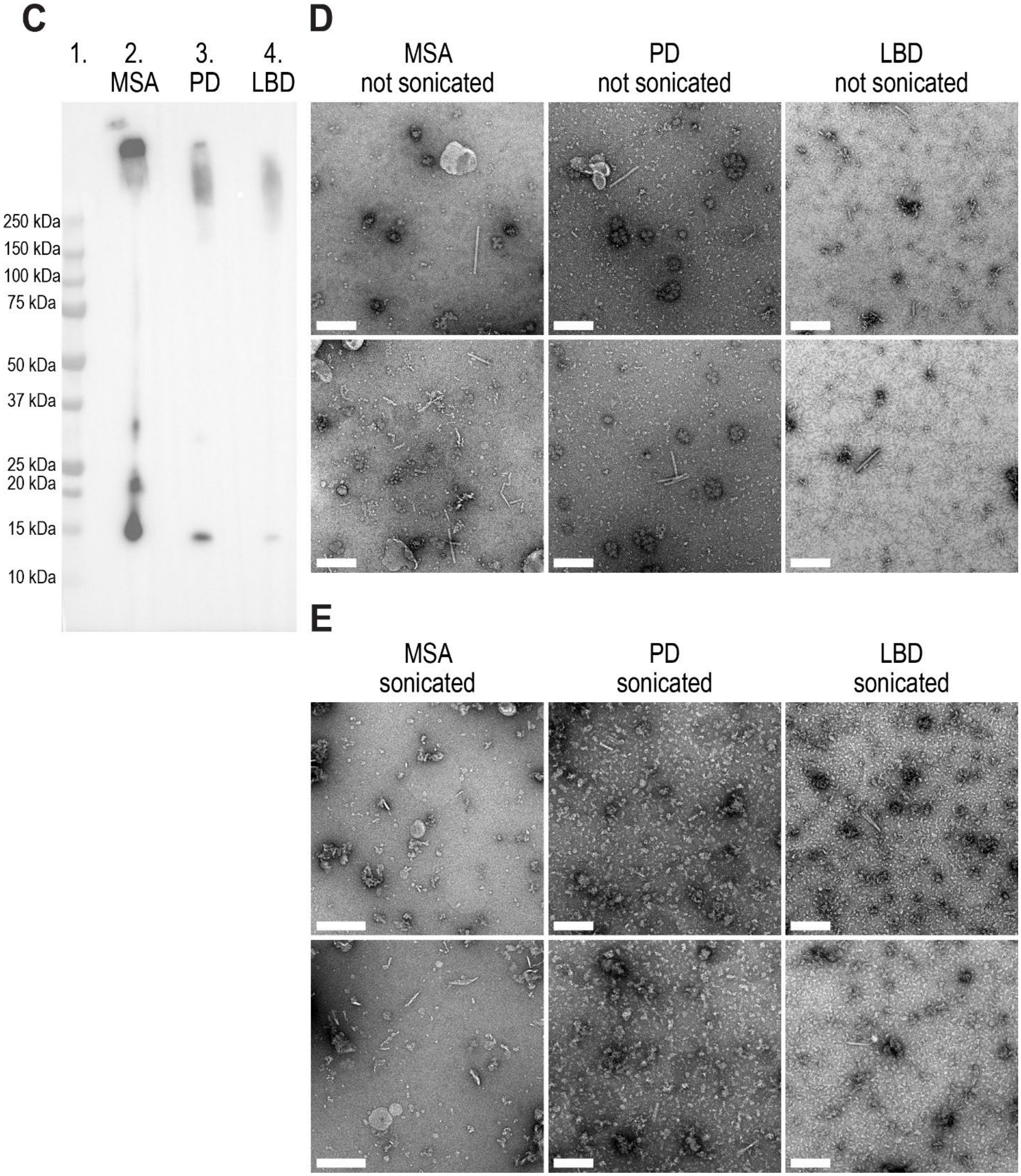

Fig. S3. Disease-linked mutations enhance aggregation of 35-110 aSyn truncation that is also responsive to seeds derived from human brain tissue. (**A**) aSyn truncations (35-110) containing disease-linked mutations (E46K, H50Q, G51D, A53T) fused to a CFP/YFP pair were overexpressed in HEK293T cells and treated with an increasing dose of the recombinant wild type aSyn fibrils to induce aggregation. Seeding efficiency was normalized to the aSyn(WT) treated with 100 nM recombinant aSyn(WT) fibrils. Error bars = standard deviation (n=3 technical replicates). (**B**) Seeding of 1, 5, 15 µg aSyn fibrils extracted from MSA, PD, LBD brain

tissue and recombinant fibrils on a panel of biosensors. Cells without treatment or treated with a vehicle (Lipofectamine2000) or negative control fibrils (100 nM tau, aβ40, aβ42 or 5 µg of sarkosyl-insoluble material from a control healthy brain) did not contain aggregates. A distinct aSyn(WT) fibril prep than in the mutagenesis screen was used as a positive control. Analysis was performed after 72 h incubation. Error bars = standard deviation (n=3 technical replicates). (**C**) Western blot analysis of sarkosyl-insoluble material from MSA (2), PD (3), and LBD (4) brain tissue. Lane (1) contains a protein ladder. (**D**) TEM images of not sonicated sarkosyl-insoluble material from MSA, PD, and LBD brain tissue. (**E**) TEM images of sonicated (20 min) sarkosyl-insoluble material from MSA, PD, and LBD brain tissue. Scale bar = 200 nm.

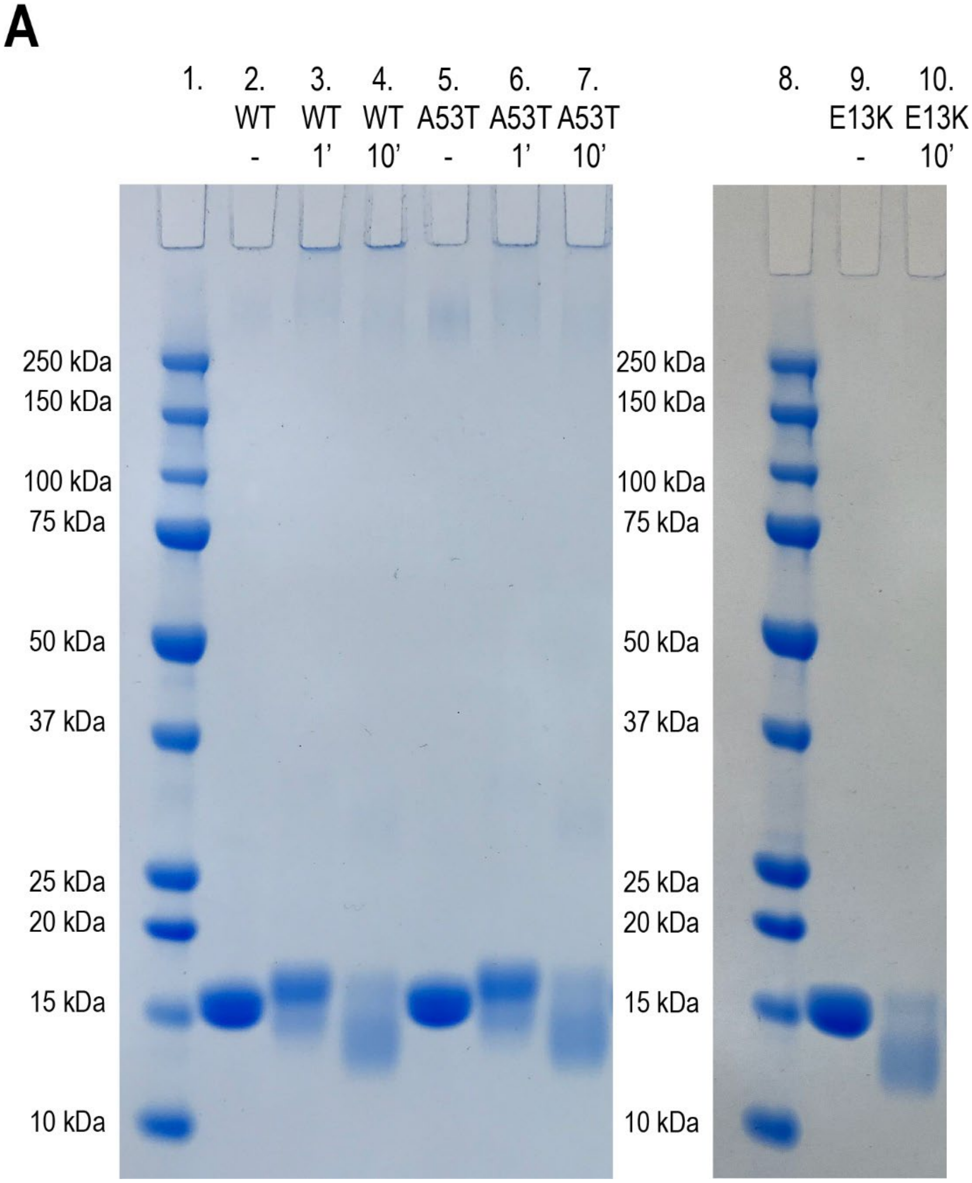

B

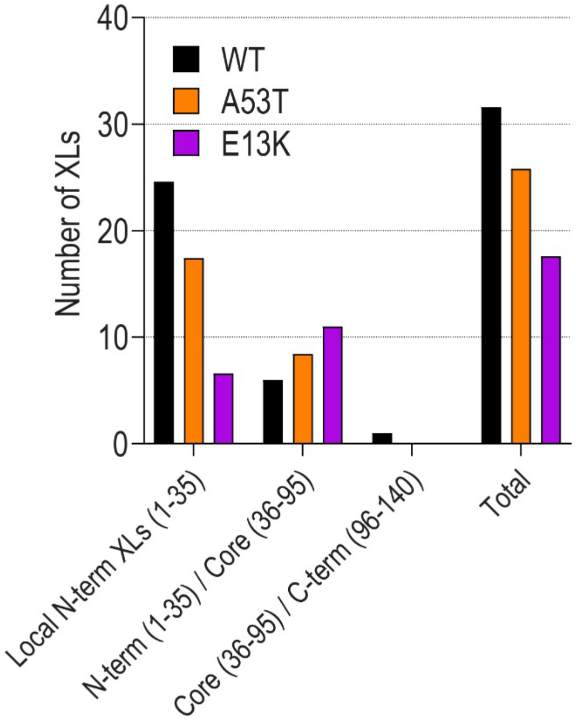

C

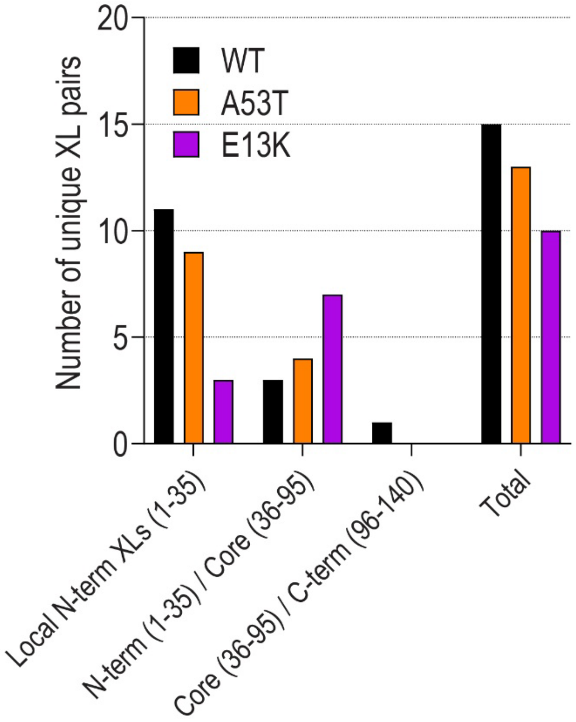

D

XL type	WT			A53T			E13K		
	pos1	pos2	mean frequency	pos1	pos2	mean frequency	pos1	pos2	mean frequency
Local N-term XL (1-35)	2	10	1				2	10	1.2
	2	12	1.6	2	12	1.8			
	2	23	1.6	2	23	1			
	6	13	1.2	10	35	1	10	20	1.8
	10	13	2	10	13	2.2			
	12	28	2	12	28	2			
	12	35	2	12	35	1			
	13	23	4.4	13	23	3.4			
	13	34	2	13	34	2			
	20	23	5	20	23	3	20	23	3.6
	23	35	1.8						
N-term (1-35) / Core (36-95)	13	45	2.6	12	57	2.4	13	61	1.4
				23	46	2.8	13	83	1.6
							21	83	1.4
	23	83	1.6	23	83	1.8	23	83	1.8
	28	60	1.8	34	83	1.4	23	61	2.8
							43	83	1
							45	83	1
Core (36-95) / C-term (96-140)	46	97	1						

E

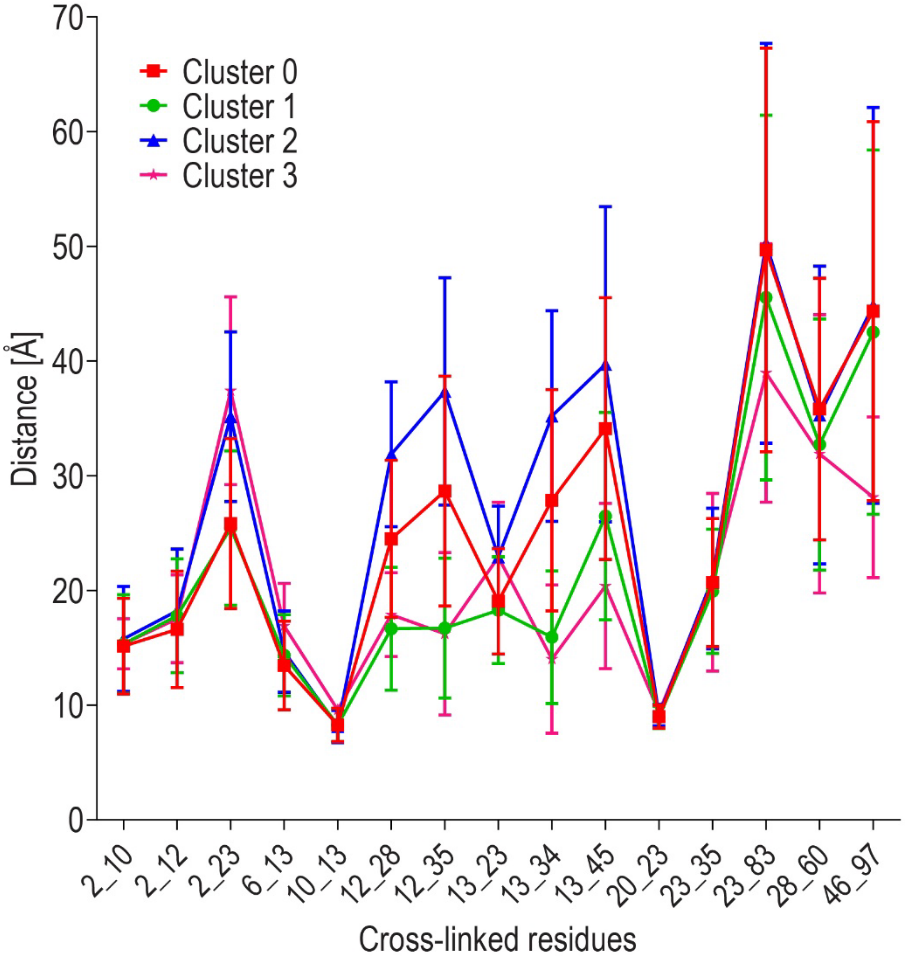

F

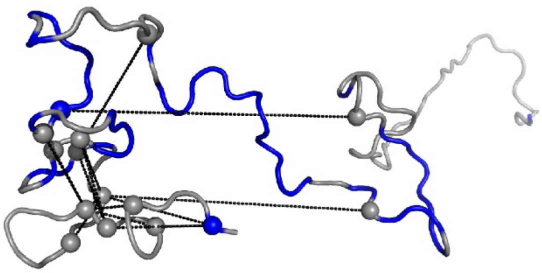

G

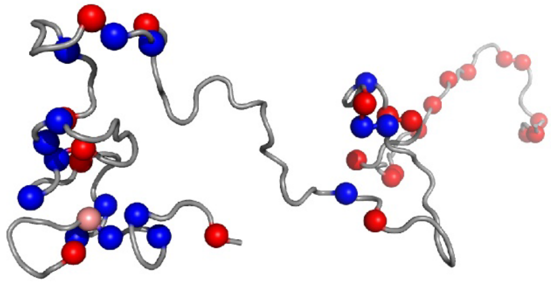

Fig. S4. E13K mutation increases aggregation propensity through an electrostatic repulsion. (**A**) SDS-PAGE Coomassie gel analysis of aSyn(WT) with no treatment (2), aSyn(WT) with DMTMM 1 min (3), aSyn(WT) with DMTMM 10 min (4), aSyn(A53T) with no treatment (5), aSyn(A53T) with DMTMM 1 min (6), aSyn(A53T) with DMTMM 10 min (7), aSyn(E13K) with no treatment (9), aSyn(E13K) with DMTMM 10 min (10) in a following buffer: 100 mM NaCl, 20 mM HEPES pH 8.0. Lanes (1) and (8) contain a protein ladder. (**B**) Bar plots showing number of unique crosslinked pairs and (**C**) total number of crosslinks in

aSyn(WT) (black), aSyn(A53T) (orange) and aSyn(E13K) (purple) within the N-terminus (aa 1-36), between the N-terminus and the aSyn core (37-95), between the core and the C-terminus (96-140) or sum from all regions (total). (**D**) A table showing all crosslinked residues in aSyn(WT), aSyn(A53T) and aSyn(E13K) (grouped by a crosslink type) along with mean frequency across 5 technical replicates. (**E**) Clustering of aSyn conformers from the Protein Ensemble Database (PED00024) (*88*). Distances between crosslinked pairs for each cluster are plotted on the graph. Cluster 1 is the most compatible with XL-MS data. Structures within that group accounts for 19.4% of all possible conformations. (**F**) An example of aSyn(WT) monomeric conformation consistent with XL-MS data. Domains are colored in blue. Alpha carbons of the crosslinked residues are shown as spheres connected with black dashed lines. (**G**) The same aSyn ensemble, but alpha carbons of positively charged residues (lysines and a histidine) are shown as blue spheres and negatively charged residues (aspartic and glutamic acids) as red spheres except glutamic acid at position 13 (E13) shown in magenta.

A

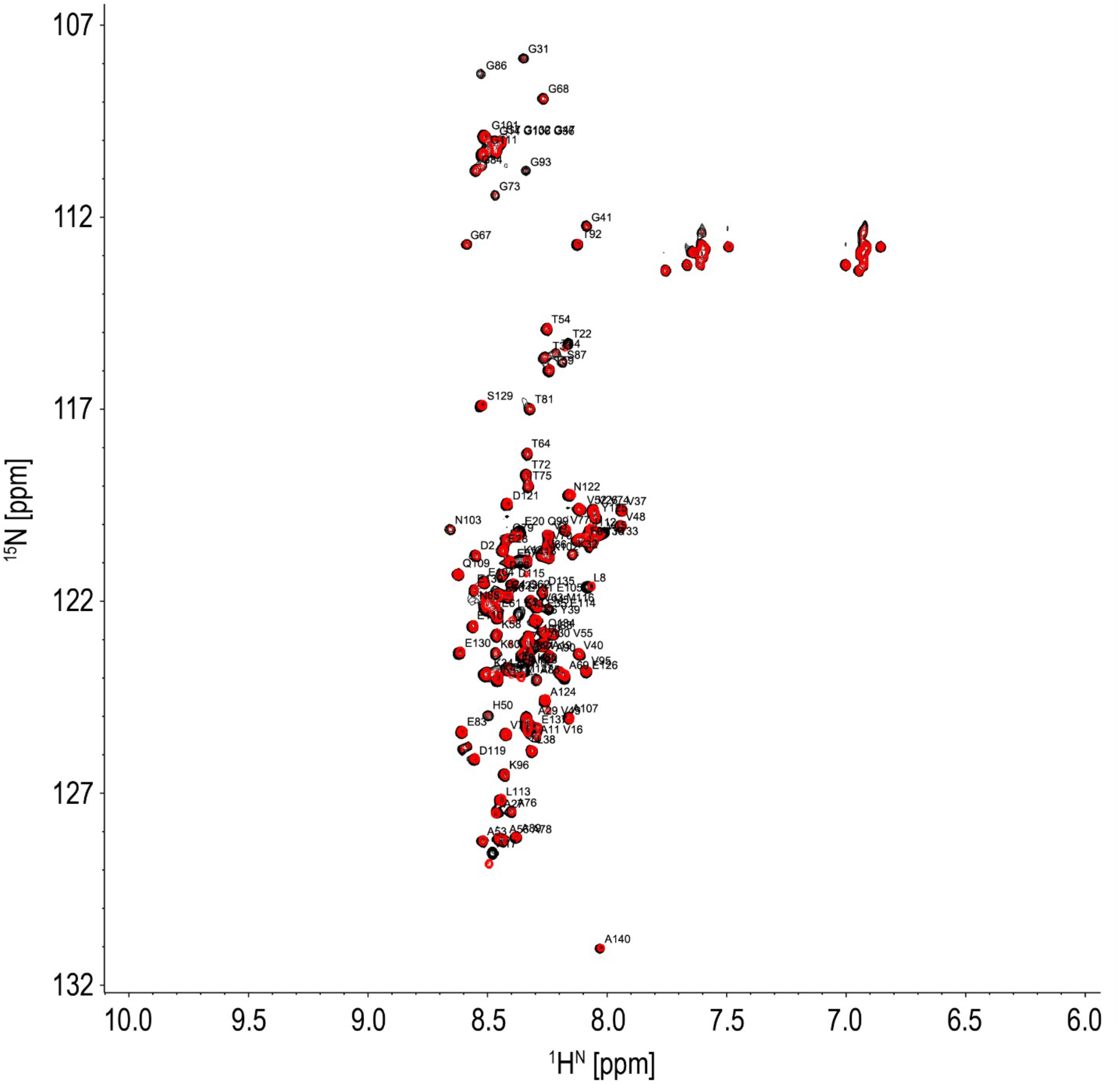

107
112
117
122
127
132
^{15}N [ppm]
10.0
9.5
9.0
8.5
8.0
7.5
7.0
6.5
6.0
^{1}H^{N} [ppm]
G31
G86
G68
G93
G73
G41
T92
G67
T54
T22
S87
S129
T81
T64
T72
T75
D121
N122
V37
V48
N103
D2
Q109
D135
L8
E114
K58
E130
V40
V55
E126
A124
H50
E83
E137
A11
V16
D119
K96
L113
A76
A53
A140

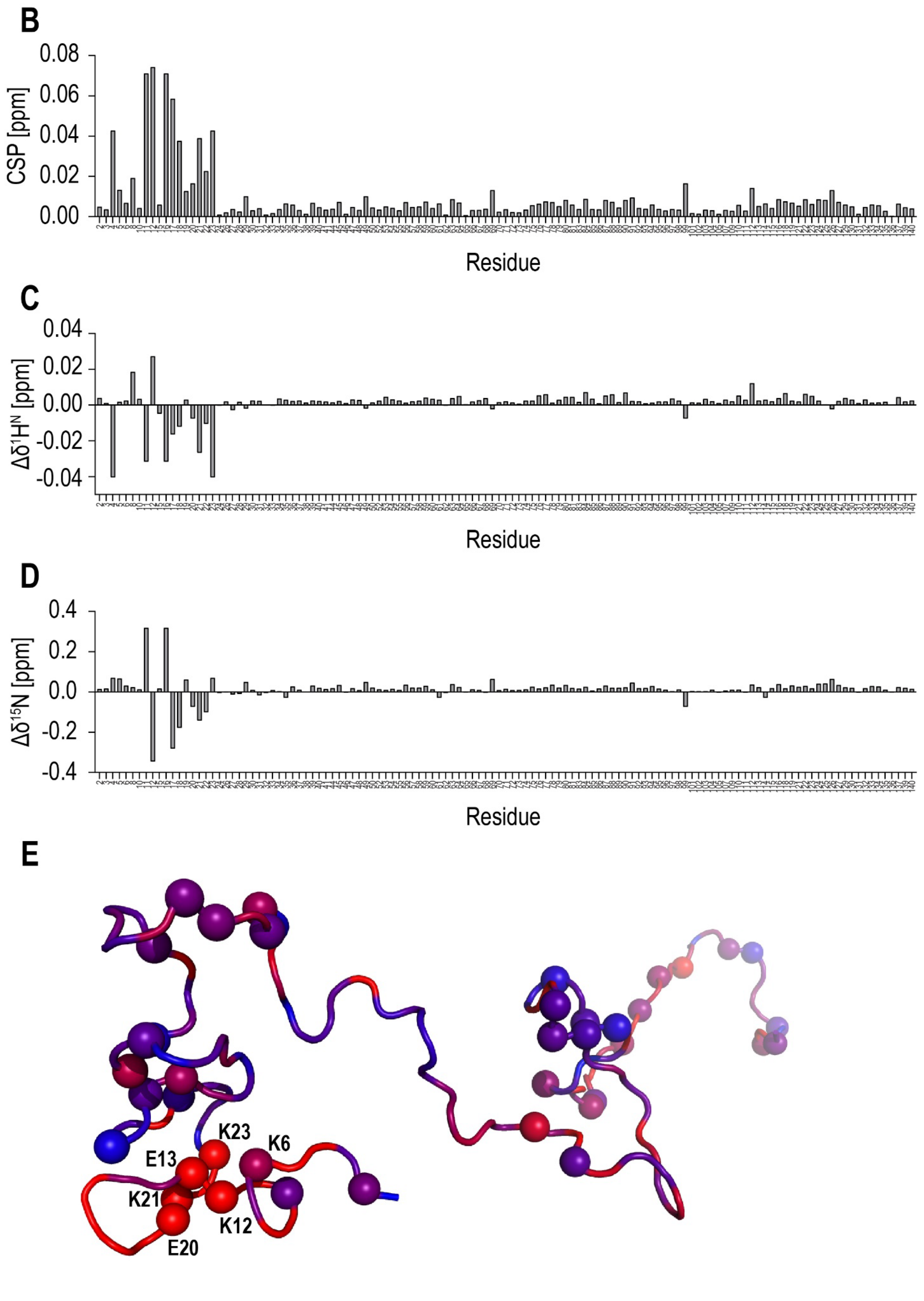
B
0.08
0.06
0.04
0.02
0.00
CSP [ppm]
Residue
C
0.04
0.02
0.00
-0.02
-0.04
Δδ¹Hᴺ [ppm]
Residue
D
0.4
0.2
0.0
-0.2
-0.4
Δδ¹⁵N [ppm]
Residue
E
K23
K6
E13
K21
K12
E20

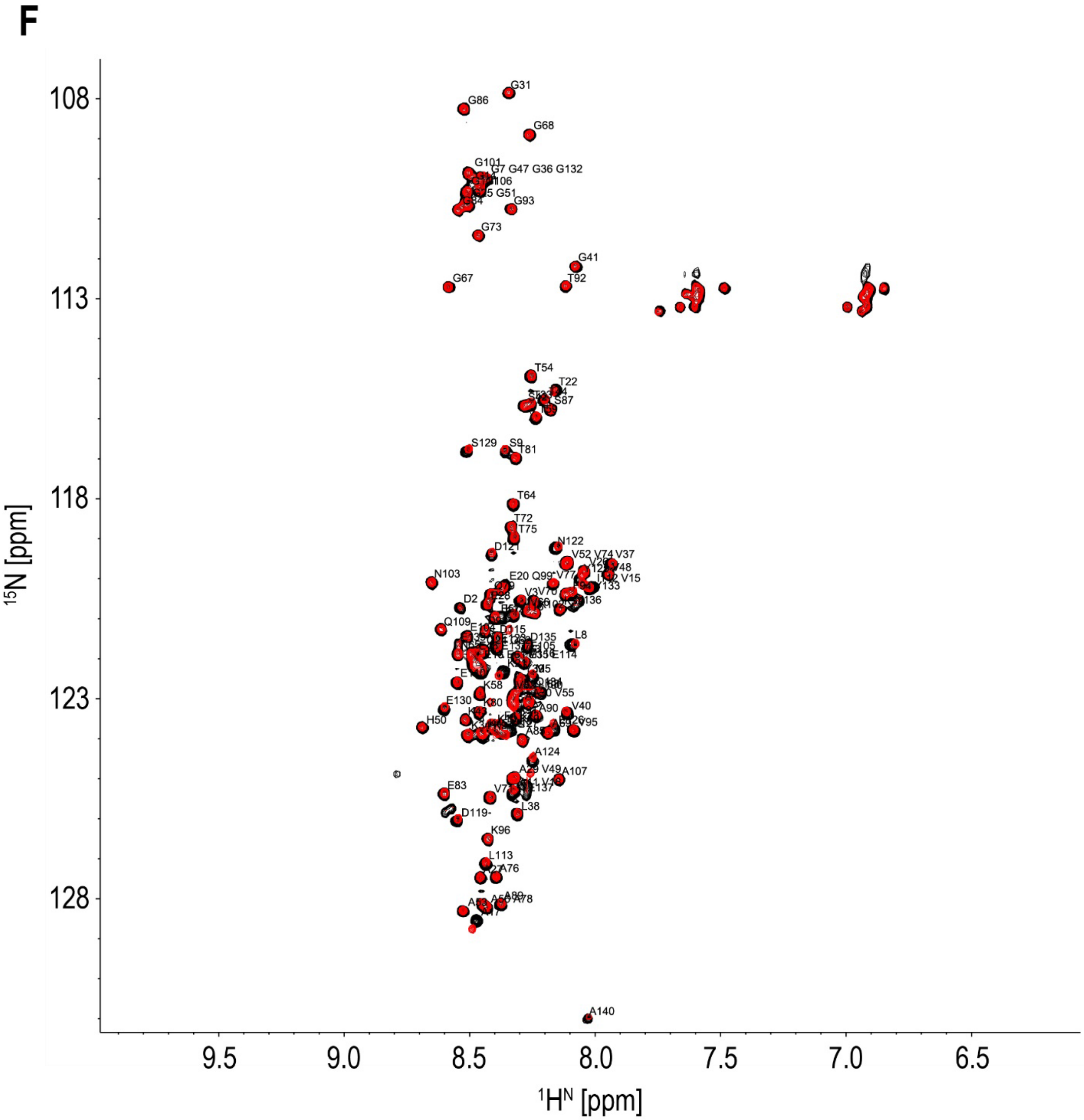
F
108
113
118
123
128
15N [ppm]
9.5
9.0
8.5
8.0
7.5
7.0
6.5
1HN [ppm]
G31
G86
G68
G101
G7 G47 G36 G132
G51
G93
G73
G41
G67
T92
T54
T22
S87
S129
S9
T81
T64
T72
T75
N122
D121
V52 V74 V37
V48
V15
N103
E20 Q99
V77
D2
Q109
D135
L8
E105
E114
K58
E130
V40
H50
A90
V55
A124
V49
A107
E83
L38
D119
K96
L113
A76
A78
A140

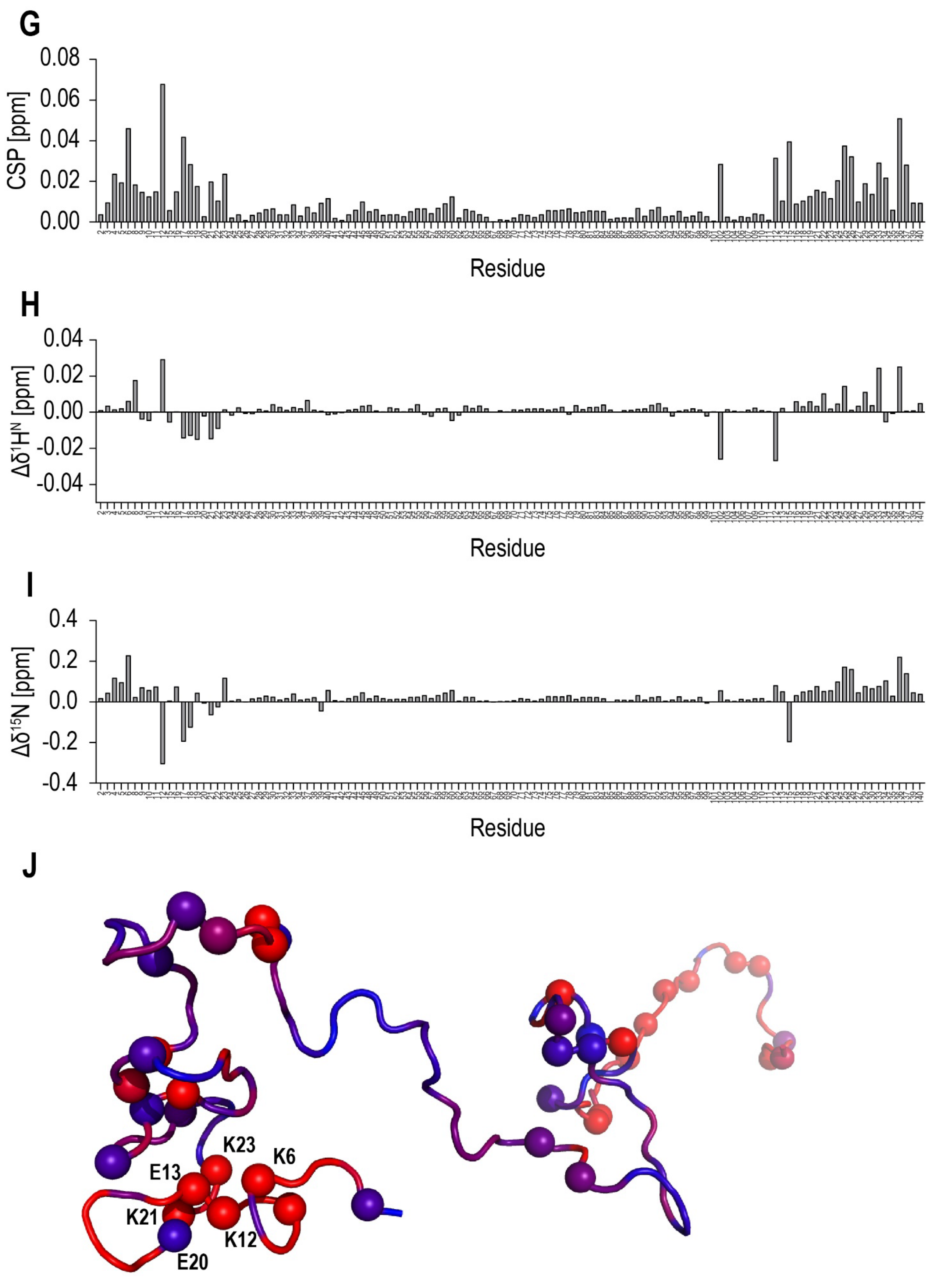

G
CSP [ppm]
0.08
0.06
0.04
0.02
0.00
Residue
H
Δδ¹Hᴺ [ppm]
0.04
0.02
0.00
-0.02
-0.04
Residue
I
Δδ¹⁵N [ppm]
0.4
0.2
0.0
-0.2
-0.4
Residue
J
K23
K6
E13
K21
K12
E20

Fig. S5. E13K perturbs the N-terminal local structure. (**A**) ^{1}H-^{15}N HSQC spectra of aSyn(WT) (black) and aSyn(E13K) (red) recorded at 15ºC and 30 µM concentration in 20 mM HEPES pH 7.4, 100 mM NaCl. (**B**) Chemical shifts perturbations (CSPs) caused by the E13K mutation in the ^{1}H-^{15}N HSQC spectra shown in Fig. 10A and S5A (formula used for calculation is described in the method section). Shifts for residues with lower assignment confidence were excluded from the graph and calculations. Average CSP = 0.00997 ppm. (**C**) Chemical shift changes for proton ($\Delta\delta^{1}H^{N}$) and (**D**) nitrogen ($\Delta\delta^{15}N$) in the ^{1}H-^{15}N HSQC spectra shown in Fig. 10A and S5A of aSyn(WT) vs. aSyn(E13K) (30 µM aSyn in 20 mM HEPES pH 7.4, 100 mM NaCl at 15ºC). Shifts for residues with lower assignment confidence were excluded from the graph and calculations. (**E**) aSyn monomeric conformation as shown previously in the Fig. S4G but colored according to the chemical shift perturbations (CSP) for aSyn at 30 µM in 20 mM HEPES pH 7.4, 100 mM NaCl at 15ºC. In red CSP > 0.00997 ppm, in blue – no change. The alpha carbons of charged residues are shown as spheres. (**F**) ^{1}H-^{15}N HSQC HSQC spectra of aSyn(WT) (black) and aSyn(E13K) (red) recorded at 15ºC and 30 µM concentration in 20 mM sodium phosphate pH 6.0. (**G**) Chemical shift perturbations (CSPs) caused by the E13K mutation in the ^{1}H-^{15}N HSQC spectra shown in Fig. S5F (formula used for calculation is described in the method section). Shifts for residues with lower assignment confidence were excluded from the graph and calculations. Average CSP = 0.011738 ppm. (**H**) Chemical shift changes for proton ($\Delta\delta^{1}H^{N}$) and (**I**) nitrogen ($\Delta\delta^{15}N$) in the ^{1}H-^{15}N HSQC spectra shown in Fig. S5F of aSyn(WT) vs. aSyn(E13K). Shifts for residues with lower assignment confidence were excluded from the graph and calculations. (**J**) aSyn monomeric conformation as shown previously in the Fig. S4G but colored according to the chemical shift perturbations (CSP) for aSyn at 30 µM in 20 mM sodium phosphate pH 6.0 at 15ºC. In red CSP > 0.011738 ppm, in blue – no change. The alpha carbons of charged residues are shown as spheres.

REFERENCES AND NOTES

1. D. D. Murphy, S. M. Rueter, J. Q. Trojanowski, V. M. Lee, Synucleins are developmentally expressed, and α-synuclein regulates the size of the presynaptic vesicular pool in primary hippocampal neurons. *J. Neurosci.* **20**, 3214–3220 (2000).

2. J. Sun, L. Wang, H. Bao, S. Premi, U. Das, E. R. Chapman, S. Roy, Functional cooperation of α-synuclein and VAMP2 in synaptic vesicle recycling. *Proc. Natl. Acad. Sci. U.S.A.* **116**, 11113–11115 (2019).

3. J. Burre, M. Sharma, T. Tsetsenis, V. Buchman, M. R. Etherton, T. C. Sudhof, α-Synuclein promotes SNARE-complex assembly in vivo and in vitro. *Science* **329**, 1663–1667 (2010).

4. M. G. Spillantini, R. A. Crowther, R. Jakes, M. Hasegawa, M. Goedert, α-Synuclein in filamentous inclusions of Lewy bodies from Parkinson's disease and dementia with Lewy bodies. *Proc. Natl. Acad. Sci. U.S.A.* **95**, 6469–6473 (1998).

5. K. Wakabayashi, M. Yoshimoto, S. Tsuji, H. Takahashi, α-Synuclein immunoreactivity in glial cytoplasmic inclusions in multiple system atrophy. *Neurosci. Lett.* **249**, 180–182 (1998).

6. J. Y. Li, E. Englund, J. L. Holton, D. Soulet, P. Hagell, A. J. Lees, T. Lashley, N. P. Quinn, S. Rehncrona, A. Bjorklund, H. Widner, T. Revesz, O. Lindvall, P. Brundin, Lewy bodies in grafted neurons in subjects with Parkinson's disease suggest host-to-graft disease propagation. *Nat. Med.* **14**, 501–503 (2008).

7. S. B. Prusiner, A. L. Woerman, D. A. Mordes, J. C. Watts, R. Rampersaud, D. B. Berry, S. Patel, A. Oehler, J. K. Lowe, S. N. Kravitz, D. H. Geschwind, D. V. Glidden, G. M. Halliday, L. T. Middleton, S. M. Gentleman, L. T. Grinberg, K. Giles, Evidence for α-synuclein prions causing multiple system atrophy in humans with parkinsonism. *Proc. Natl. Acad. Sci. U.S.A.* **112**, E5308–E5317 (2015).

8. J. I. Ayers, J. Lee, O. Monteiro, A. L. Woerman, A. A. Lazar, C. Condello, N. A. Paras, S. B. Prusiner, Different α-synuclein prion strains cause dementia with Lewy bodies and multiple system atrophy. *Proc. Natl. Acad. Sci. U.S.A.* **119**, e2113489119 (2022).

9. J. F. Reyes, N. L. Rey, L. Bousset, R. Melki, P. Brundin, E. Angot, α-Synuclein transfers from neurons to oligodendrocytes. *Glia* **62**, 387–398 (2014).

10. C. Hansen, E. Angot, A. L. Bergstrom, J. A. Steiner, L. Pieri, G. Paul, T. F. Outeiro, R. Melki, P. Kallunki, K. Fog, J. Y. Li, P. Brundin, α-Synuclein propagates from mouse brain to grafted dopaminergic neurons and seeds aggregation in cultured human cells. *J. Clin. Invest.* **121**, 715–725 (2011).

11. A. L. Woerman, J. Stohr, A. Aoyagi, R. Rampersaud, Z. Krejciova, J. C. Watts, T. Ohyama, S. Patel, K. Widjaja, A. Oehler, D. W. Sanders, M. I. Diamond, W. W. Seeley, L. T. Middleton, S. M. Gentleman, D. A. Mordes, T. C. Sudhof, K. Giles, S. B. Prusiner, Propagation of prions causing synucleinopathies in cultured cells. *Proc. Natl. Acad. Sci. U.S.A.* **112**, E4949–E4958 (2015).

12. T. R. Yamasaki, B. B. Holmes, J. L. Furman, D. D. Dhavale, B. W. Su, E. S. Song, N. J. Cairns, P. T. Kotzbauer, M. I. Diamond, Parkinson's disease and multiple system atrophy have distinct α-synuclein seed characteristics. *J. Biol. Chem.* **294**, 1045–1058 (2019).

13. B. B. Holmes, J. L. Furman, T. E. Mahan, T. R. Yamasaki, H. Mirbaha, W. C. Eades, L. Belaygorod, N. J. Cairns, D. M. Holtzman, M. I. Diamond, Proteopathic tau seeding predicts tauopathy in vivo. *Proc. Natl. Acad. Sci. U.S.A.* **111**, E4376–E4385 (2014).

14. M. H. Polymeropoulos, C. Lavedan, E. Leroy, S. E. Ide, A. Dehejia, A. Dutra, B. Pike, H. Root, J. Rubenstein, R. Boyer, E. S. Stenroos, S. Chandrasekharappa, A. Athanassiadou, T. Papapetropoulos, W. G. Johnson, A. M. Lazzarini, R. C. Duvoisin, G. Di Iorio, L. I. Golbe, R. L. Nussbaum, Mutation in the α-synuclein gene identified in families with Parkinson's disease. *Science* **276**, 2045–2047 (1997).

15. R. Kruger, W. Kuhn, T. Muller, D. Woitalla, M. Graeber, S. Kosel, H. Przuntek, J. T. Epplen, L. Schols, O. Riess, Ala30Pro mutation in the gene encoding α-synuclein in Parkinson's disease. *Nat. Genet.* **18**, 106–108 (1998).

16. J. J. Zarranz, J. Alegre, J. C. Gomez-Esteban, E. Lezcano, R. Ros, I. Ampuero, L. Vidal, J. Hoenicka, O. Rodriguez, B. Atares, V. Llorens, E. Gomez Tortosa, T. del Ser, D. G. Munoz, J. G. de Yebenes, The new mutation, E46K, of α-synuclein causes Parkinson and Lewy body dementia. *Ann. Neurol.* **55**, 164–173 (2004).

17. S. Appel-Cresswell, C. Vilarino-Guell, M. Encarnacion, H. Sherman, I. Yu, B. Shah, D. Weir, C. Thompson, C. Szu-Tu, J. Trinh, J. O. Aasly, A. Rajput, A. H. Rajput, A. Jon Stoessl, M. J. Farrer, α-Synuclein p.H50Q, a novel pathogenic mutation for Parkinson's disease. *Mov. Disord.* **28**, 811–813 (2013).

18. S. Lesage, M. Anheim, F. Letournel, L. Bousset, A. Honore, N. Rozas, L. Pieri, K. Madiona, A. Durr, R. Melki, C. Verny, A. Brice, G. G51D α-synuclein mutation causes a novel parkinsonian-pyramidal syndrome. *Ann. Neurol.* **73**, 459–471 (2013).

19. P. Pasanen, L. Myllykangas, M. Siitonen, A. Raunio, S. Kaakkola, J. Lyytinen, P. J. Tienari, M. Poyhonen, A. Paetau, Novel α-synuclein mutation A53E associated with atypical multiple system atrophy and Parkinson's disease-type pathology. *Neurobiol. Aging* **35**, 2180.e1–2180.e5 (2014).

20. H. Yoshino, M. Hirano, A. J. Stoessl, Y. Imamichi, A. Ikeda, Y. Li, M. Funayama, I. Yamada, Y. Nakamura, V. Sossi, M. J. Farrer, K. Nishioka, N. Hattori, Homozygous α-synuclein p.A53V in familial Parkinson's disease. *Neurobiol. Aging* **57**, 248.e7–248.e12 (2017).

21. A. Kapasi, J. R. Brosch, K. N. Nudelman, S. Agrawal, T. M. Foroud, J. A. Schneider, A novel *SNCA* E83Q mutation in a case of dementia with Lewy bodies and atypical frontotemporal lobar degeneration. *Neuropathology* **40**, 620–626 (2020).

22. K. Uéda, H. Fukushima, E. Masliah, Y. Xia, A. Iwai, M. Yoshimoto, D. A. Otero, J. Kondo, Y. Ihara, T. Saitoh, Molecular cloning of cDNA encoding an unrecognized component of amyloid in Alzheimer disease. *Proc. Natl. Acad. Sci. U.S.A.* **90**, 11282–11286 (1993).

23. A. Iwai, E. Masliah, M. Yoshimoto, N. Ge, L. Flanagan, H. A. de Silva, A. Kittel, T. Saitoh, The precursor protein of non-Aβ component of Alzheimer's disease amyloid is a presynaptic protein of the central nervous system. *Neuron* **14**, 467–475 (1995).

24. K. Nienhaus, G. U. Nienhaus, J. Wiedenmann, H. Nar, Structural basis for photo-induced protein cleavage and green-to-red conversion of fluorescent protein EosFP. *Proc. Natl. Acad. Sci. U.S.A.* **102**, 9156–9159 (2005).

25. T. Khan, T. S. Kandola, J. Wu, S. Venkatesan, E. Ketter, J. J. Lange, A. Rodriguez Gama, A. Box, J. R. Unruh, M. Cook, R. Halfmann, Quantifying nucleation in vivo reveals the physical basis of prion-like phase behavior. *Mol. Cell* **71**, 155–168.e7 (2018).

26. M. B. Fares, N. Ait-Bouziad, I. Dikiy, M. K. Mbefo, A. Jovicic, A. Kiely, J. L. Holton, S. J. Lee, A. D. Gitler, D. Eliezer, H. A. Lashuel, The novel Parkinson's disease linked mutation G51D attenuates in vitro aggregation and membrane binding of α-synuclein, and enhances its secretion and nuclear localization in cells. *Hum. Mol. Genet.* **23**, 4491–4509 (2014).

27. D. Ghosh, S. Sahay, P. Ranjan, S. Salot, G. M. Mohite, P. K. Singh, S. Dwivedi, E. Carvalho, R. Banerjee, A. Kumar, S. K. Maji, The newly discovered Parkinson's disease associated Finnish mutation (A53E) attenuates α-synuclein aggregation and membrane binding. *Biochemistry* **53**, 6419–6421 (2014).

28. O. Khalaf, B. Fauvet, A. Oueslati, I. Dikiy, A. L. Mahul-Mellier, F. S. Ruggeri, M. K. Mbefo, F. Vercruysse, G. Dietler, S. J. Lee, D. Eliezer, H. A. Lashuel, The H50Q mutation enhances α-synuclein aggregation, secretion, and toxicity. *J. Biol. Chem.* **289**, 21856–21876 (2014).

29. G. M. Mohite, R. Kumar, R. Panigrahi, A. Navalkar, N. Singh, D. Datta, S. Mehra, S. Ray, L. G. Gadhe, S. Das, N. Singh, D. Chatterjee, A. Kumar, S. K. Maji, Comparison of kinetics, toxicity, oligomer formation, and membrane binding capacity of α-synuclein familial mutations at the A53 site, including the newly discovered a53v mutation. *Biochemistry* **57**, 5183–5187 (2018).

30. S. T. Kumar, A. L. Mahul-Mellier, R. N. Hegde, G. Riviere, R. Moons, A. Ibáñez de Opakua, P. Magalhaes, I. Rostami, S. Donzelli, F. Sobott, M. Zweckstetter, H. A. Lashuel, A NAC domain mutation (E83Q) unlocks the pathogenicity of human α-synuclein and recapitulates its pathological diversity. *Sci. Adv.* **8**, eabn0044 (2022).

31. T. J. van Ham, A. Esposito, J. R. Kumita, S. T. Hsu, G. S. Kaminski Schierle, C. F. Kaminski, C. M. Dobson, E. A. Nollen, C. W. Bertoncini, Towards multiparametric fluorescent imaging of amyloid formation: Studies of a YFP model of α-synuclein aggregation. *J. Mol. Biol.* **395**, 627–642 (2010).

32. V. Mullapudi, J. Vaquer-Alicea, V. Bommareddy, A. R. Vega, B. D. Ryder, C. L. White, 3rd, M. I. Diamond, L. A. Joachimiak, Network of hotspot interactions cluster tau amyloid folds. *Nat. Commun.* **14**, 895 (2023).

33. J. H. Xue, D. M. Titterington, *t* Tests, F tests and Otsu's methods for image thresholding. *IEEE Trans. Image Process.* **20**, 2392–2396 (2011).

34. M. D. Tuttle, G. Comellas, A. J. Nieuwkoop, D. J. Covell, D. A. Berthold, K. D. Kloepper, J. M. Courtney, J. K. Kim, A. M. Barclay, A. Kendall, W. Wan, G. Stubbs, C. D. Schwieters, V. M. Lee, J. M. George, C. M. Rienstra, Solid-state NMR structure of a pathogenic fibril of full-length human α-synuclein. *Nat. Struct. Mol. Biol.* **23**, 409–415 (2016).

35. B. Li, P. Ge, K. A. Murray, P. Sheth, M. Zhang, G. Nair, M. R. Sawaya, W. S. Shin, D. R. Boyer, S. Ye, D. S. Eisenberg, Z. H. Zhou, L. Jiang, Cryo-EM of full-length α-synuclein reveals fibril polymorphs with a common structural kernel. *Nat. Commun.* **9**, 3609 (2018).

36. Y. Li, C. Zhao, F. Luo, Z. Liu, X. Gui, Z. Luo, X. Zhang, D. Li, C. Liu, X. Li, Amyloid fibril structure of α-synuclein determined by cryo-electron microscopy. *Cell Res.* **28**, 897–903 (2018).

37. K. Zhao, Y. J. Lim, Z. Liu, H. Long, Y. Sun, J. J. Hu, C. Zhao, Y. Tao, X. Zhang, D. Li, Y. M. Li, C. Liu, Parkinson's disease-related phosphorylation at Tyr^{39} rearranges α-synuclein

amyloid fibril structure revealed by cryo-EM. *Proc. Natl. Acad. Sci. U.S.A.* **117**, 20305–20315 (2020).

38. D. R. Boyer, B. Li, C. Sun, W. Fan, M. R. Sawaya, L. Jiang, D. S. Eisenberg, Structures of fibrils formed by α-synuclein hereditary disease mutant H50Q reveal new polymorphs. *Nat. Struct. Mol. Biol.* **26**, 1044–1052 (2019).

39. D. R. Boyer, B. Li, C. Sun, W. Fan, K. Zhou, M. P. Hughes, M. R. Sawaya, L. Jiang, D. S. Eisenberg, The α-synuclein hereditary mutation E46K unlocks a more stable, pathogenic fibril structure. *Proc. Natl. Acad. Sci. U.S.A.* **117**, 3592–3602 (2020).

40. Y. Sun, S. Hou, K. Zhao, H. Long, Z. Liu, J. Gao, Y. Zhang, X. D. Su, D. Li, C. Liu, Cryo-EM structure of full-length α-synuclein amyloid fibril with Parkinson's disease familial A53T mutation. *Cell Res.* **30**, 360–362 (2020).

41. K. Zhao, Y. Li, Z. Liu, H. Long, C. Zhao, F. Luo, Y. Sun, Y. Tao, X. D. Su, D. Li, X. Li, C. Liu, Parkinson's disease associated mutation E46K of α-synuclein triggers the formation of a distinct fibril structure. *Nat. Commun.* **11**, 2643 (2020).

42. M. Schweighauser, Y. Shi, A. Tarutani, F. Kametani, A. G. Murzin, B. Ghetti, T. Matsubara, T. Tomita, T. Ando, K. Hasegawa, S. Murayama, M. Yoshida, M. Hasegawa, S. H. W. Scheres, M. Goedert, Structures of α-synuclein filaments from multiple system atrophy. *Nature* **585**, 464–469 (2020).

43. Y. Yang, Y. Shi, M. Schweighauser, X. Zhang, A. Kotecha, A. G. Murzin, H. J. Garringer, P. W. Cullinane, Y. Saito, T. Foroud, T. T. Warner, K. Hasegawa, R. Vidal, S. Murayama, T. Revesz, B. Ghetti, M. Hasegawa, T. Lashley, S. H. W. Scheres, M. Goedert, Structures of α-synuclein filaments from human brains with Lewy pathology. *Nature* **610**, 791–795 (2022).

44. R. Guerrero-Ferreira, N. M. Taylor, D. Mona, P. Ringler, M. E. Lauer, R. Riek, M. Britschgi, H. Stahlberg, Cryo-EM structure of α-synuclein fibrils. *eLife* **7**, e36402 (2018).

45. X. Ni, R. P. McGlinchey, J. Jiang, J. C. Lee, Structural insights into α-Synuclein fibril polymorphism: Effects of parkinson's disease-related C-terminal truncations. *J. Mol. Biol.* **431**, 3913–3919 (2019).

46. R. P. McGlinchey, X. Ni, J. A. Shadish, J. Jiang, J. C. Lee, The N terminus of α-synuclein dictates fibril formation. *Proc. Natl. Acad. Sci. U.S.A.* **118** (2021).

47. I. V. Murray, B. I. Giasson, S. M. Quinn, V. Koppaka, P. H. Axelsen, H. Ischiropoulos, J. Q. Trojanowski, V. M. Lee, Role of α-synuclein carboxy-terminus on fibril formation in vitro. *Biochemistry* **42**, 8530–8540 (2003).

48. Z. A. Sorrentino, N. Vijayaraghavan, K. M. Gorion, C. J. Riffe, K. H. Strang, J. Caldwell, B. I. Giasson, Physiological C-terminal truncation of α-synuclein potentiates the prion-like formation of pathological inclusions. *J. Biol. Chem.* **293**, 18914–18932 (2018).

49. C. Peng, R. J. Gathagan, D. J. Covell, C. Medellin, A. Stieber, J. L. Robinson, B. Zhang, R. M. Pitkin, M. F. Olufemi, K. C. Luk, J. Q. Trojanowski, V. M. Lee, Cellular milieu imparts distinct pathological α-synuclein strains in α-synucleinopathies. *Nature* **557**, 558–563 (2018).

50. H. Mirbaha, D. Chen, O. A. Morazova, K. M. Ruff, A. M. Sharma, X. Liu, M. Goodarzi, R. V. Pappu, D. W. Colby, H. Mirzaei, L. A. Joachimiak, M. I. Diamond, Inert and seed-competent tau monomers suggest structural origins of aggregation. *eLife* **7**, e36584 (2018).

51. A. Leitner, L. A. Joachimiak, P. Unverdorben, T. Walzthoeni, J. Frydman, F. Forster, R. Aebersold, Chemical cross-linking/mass spectrometry targeting acidic residues in proteins and protein complexes. *Proc. Natl. Acad. Sci. U.S.A.* **111**, 9455–9460 (2014).

52. A. S. Maltsev, J. Ying, A. Bax, Impact of N-terminal acetylation of α-synuclein on its random coil and lipid binding properties. *Biochemistry* **51**, 5004–5013 (2012).

53. F. Rousseau, L. Serrano, J. W. Schymkowitz, How evolutionary pressure against protein aggregation shaped chaperone specificity. *J. Mol. Biol.* **355**, 1037–1047 (2006).

54. G. De Baets, J. Van Durme, F. Rousseau, J. Schymkowitz, A genome-wide sequence-structure analysis suggests aggregation gatekeepers constitute an evolutionary constrained functional class. *J. Mol. Biol.* **426**, 2405–2412 (2014).

55. B. I. Giasson, I. V. Murray, J. Q. Trojanowski, V. M. Lee, A hydrophobic stretch of 12 amino acid residues in the middle of α-synuclein is essential for filament assembly. *J. Biol. Chem.* **276**, 2380–2386 (2001).

56. N. Sanchez de Groot, I. Pallares, F. X. Aviles, J. Vendrell, S. Ventura, Prediction of "hot spots" of aggregation in disease-linked polypeptides. *BMC Struct. Biol.* **5**, 18 (2005).

57. A. T. Marvian, F. Aliakbari, H. Mohammad-Beigi, Z. A. Ahmadi, S. Mehrpooyan, F. Lermyte, M. Nasouti, J. F. Collingwood, D. E. Otzen, D. Morshedi, The status of the terminal regions of α-synuclein in different forms of aggregates during fibrillization. *Int. J. Biol. Macromol.* **155**, 543–550 (2020).

58. A. Khammari, S. S. Arab, M. R. Ejtehadi, The hot sites of α-synuclein in amyloid fibril formation. *Sci. Rep.* **10**, 12175 (2020).

59. R. W. Newberry, J. T. Leong, E. D. Chow, M. Kampmann, W. F. DeGrado, Deep mutational scanning reveals the structural basis for α-synuclein activity. *Nat. Chem. Biol.* **16**, 653–659 (2020).

60. L. van der Maaten, G. Hinton, Visualizing data using t-SNE. *J. Mach. Learn. Res.* **9**, 2579–2605 (2008).

61. G. C. Linderman, S. Steinerberger, Clustering with t-SNE, provably. *SIAM J. Math. Data Sci* **1**, 313–332 (2019).

62. S. Lövestam, M. Schweighauser, T. Matsubara, S. Murayama, T. Tomita, T. Ando, K. Hasegawa, M. Yoshida, A. Tarutani, M. Hasegawa, M. Goedert, S. H. W. Scheres, Seeded assembly in vitro does not replicate the structures of α-synuclein filaments from multiple system atrophy. *FEBS Open Bio* **11**, 999–1013 (2021).

63. D. Chen, K. W. Drombosky, Z. Hou, L. Sari, O. M. Kashmer, B. D. Ryder, V. A. Perez, D. R. Woodard, M. M. Lin, M. I. Diamond, L. A. Joachimiak, Tau local structure shields an amyloid-forming motif and controls aggregation propensity. *Nat. Commun.* **10**, 2493 (2019).

64. S. Lövestam, F. A. Koh, B. van Knippenberg, A. Kotecha, A. G. Murzin, M. Goedert, S. H. W. Scheres, Assembly of recombinant tau into filaments identical to those of Alzheimer's disease and chronic traumatic encephalopathy. *eLife* **11**, e76494 (2022).

65. A. D. Stephens, M. Zacharopoulou, R. Moons, G. Fusco, N. Seetaloo, A. Chiki, P. J. Woodhams, I. Mela, H. A. Lashuel, J. J. Phillips, A. De Simone, F. Sobott, G. S. K. Schierle, Extent of N-terminus exposure of monomeric α-synuclein determines its aggregation propensity. *Nat. Commun.* **11**, 2820 (2020).

66. R. Bussell, Jr., D. Eliezer, Residual structure and dynamics in Parkinson's disease-associated mutants of α-synuclein. *J. Biol. Chem.* **276**, 45996–46003 (2001).

67. M. M. Dedmon, K. Lindorff-Larsen, J. Christodoulou, M. Vendruscolo, C. M. Dobson, Mapping long-range interactions in α-synuclein using spin-label NMR and ensemble molecular dynamics simulations. *J. Am. Chem. Soc.* **127**, 476–477 (2005).

68. D. Ubbiali, M. Fratini, L. Piersimoni, C. H. Ihling, M. Kipping, I. Heilmann, C. Iacobucci, A. Sinz, Direct observation of "elongated" conformational states in α-synuclein upon liquid-liquid phase separation. *Angew. Chem. Int. Ed. Engl.* **61**, e202205726 (2022).

69. N. I. Brodie, K. A. Makepeace, E. V. Petrotchenko, C. H. Borchers, Isotopically-coded short-range hetero-bifunctional photo-reactive crosslinkers for studying protein structure. *J. Proteomics* **118**, 12–20 (2015).

70. N. I. Brodie, E. V. Petrotchenko, C. H. Borchers, The novel isotopically coded short-range photo-reactive crosslinker 2,4,6-triazido-1,3,5-triazine (TATA) for studying protein structures. *J. Proteomics* **149**, 69–76 (2016).

71. N. I. Brodie, K. I. Popov, E. V. Petrotchenko, N. V. Dokholyan, C. H. Borchers, Conformational ensemble of native α-synuclein in solution as determined by short-distance

crosslinking constraint-guided discrete molecular dynamics simulations. *PLOS Comput. Biol.* **15**, e1006859 (2019).

72. R. Porcari, C. Proukakis, C. A. Waudby, B. Bolognesi, P. P. Mangione, J. F. Paton, S. Mullin, L. D. Cabrita, A. Penco, A. Relini, G. Verona, M. Vendruscolo, M. Stoppini, G. G. Tartaglia, C. Camilloni, J. Christodoulou, A. H. Schapira, V. Bellotti, The H50Q mutation induces a 10-fold decrease in the solubility of α-synuclein. *J. Biol. Chem.* **290**, 2395–2404 (2015).

73. D. Fischer, M. D. Mukrasch, M. von Bergen, A. Klos-Witkowska, J. Biernat, C. Griesinger, E. Mandelkow, M. Zweckstetter, Structural and microtubule binding properties of tau mutants of frontotemporal dementias. *Biochemistry* **46**, 2574–2582 (2007).

74. J. Bai, K. Cheng, M. Liu, C. Li, Impact of the α-synuclein initial ensemble structure on fibrillation pathways and kinetics. *J. Phys. Chem. B* **120**, 3140–3147 (2016).

75. D. Eliezer, E. Kutluay, R. Bussell, Jr., G. Browne, Conformational properties of α-synuclein in its free and lipid-associated states. *J. Mol. Biol.* **307**, 1061–1073 (2001).

76. J. L. Furman, B. B. Holmes, M. I. Diamond, Sensitive detection of proteopathic seeding activity with FRET flow cytometry. *J. Vis. Exp.*, **106**, e53205 (2015).

77. J. M. Gaspar, NGmerge: Merging paired-end reads via novel empirically-derived models of sequencing errors. *BMC Bioinformatics* **19**, 536 (2018).

78. D. Chen, L. A. Joachimiak, Cross-linking mass spectrometry analysis of metastable compact structures in intrinsically disordered proteins. *Methods Mol. Biol.* **2551**, 189–201 (2023).

79. F. Delaglio, S. Grzesiek, G. W. Vuister, G. Zhu, J. Pfeifer, A. Bax, NMRPipe: A multidimensional spectral processing system based on UNIX pipes. *J. Biomol. NMR* **6**, 277–293 (1995).

80. B. A. Johnson, R. A. Blevins, NMR View: NMR view: A computer program for the visualization and analysis of NMR data. *J. Biomol. NMR* **4**, 603–614 (1994).

81. R. Heim, R. Y. Tsien, Engineering green fluorescent protein for improved brightness, longer wavelengths and fluorescence resonance energy transfer. *Curr. Biol.* **6**, 178–182 (1996).

82. M. Ormo, A. B. Cubitt, K. Kallio, L. A. Gross, R. Y. Tsien, S. J. Remington, Crystal structure of the *Aequorea victoria* green fluorescent protein. *Science* **273**, 1392–1395 (1996).

83. M. Zhang, H. Chang, Y. Zhang, J. Yu, L. Wu, W. Ji, J. Chen, B. Liu, J. Lu, Y. Liu, J. Zhang, P. Xu, T. Xu, Rational design of true monomeric and bright photoactivatable fluorescent proteins. *Nat. Methods* **9**, 727–729 (2012).

84. R. Guerrero-Ferreira, N. M. Taylor, A. A. Arteni, P. Kumari, D. Mona, P. Ringler, M. Britschgi, M. E. Lauer, A. Makky, J. Verasdonck, R. Riek, R. Melki, B. H. Meier, A. Bockmann, L. Bousset, H. Stahlberg, Two new polymorphic structures of human full-length α-synuclein fibrils solved by cryo-electron microscopy. *eLife* **8** (2019).

85. Y. Sun, H. Long, W. Xia, K. Wang, X. Zhang, B. Sun, Q. Cao, Y. Zhang, B. Dai, D. Li, C. Liu, The hereditary mutation G51D unlocks a distinct fibril strain transmissible to wild-type α-synuclein. *Nat. Commun.* **12**, 6252 (2021).

86. Y. Fan, Y. Sun, W. Yu, Y. Tao, W. Xia, Y. Liu, Q. Zhao, Y. Tang, Y. Sun, F. Liu, Q. Cao, J. Wu, C. Liu, J. Wang, D. Li, Conformational change of α-synuclein fibrils in cerebrospinal fluid from different clinical phases of Parkinson's disease. *Structure* **31**, 78–87.e5 (2023).

87. B. Frieg, L. Antonschmidt, C. Dienemann, J. A. Geraets, E. E. Najbauer, D. Matthes, B. L. de Groot, L. B. Andreas, S. Becker, C. Griesinger, G. F. Schroder, The 3D structure of lipidic fibrils of α-synuclein. *Nat. Commun.* **13**, 6810 (2022).
